# 25-hydroxycholesterol contributes to cerebral inflammation of X-linked adrenoleukodystrophy through activation of the NLRP3 inflammasome

**DOI:** 10.1038/ncomms13129

**Published:** 2016-10-25

**Authors:** Jiho Jang, Sangjun Park, Hye Jin Hur, Hyun-Ju Cho, Inhwa Hwang, Yun Pyo Kang, Isak Im, Hyunji Lee, Eunju Lee, Wonsuk Yang, Hoon-Chul Kang, Sung Won Kwon, Je-Wook Yu, Dong-Wook Kim

**Affiliations:** 1 Department of Physiology and Brain Korea 21 PLUS Project for Medical Science, Yonsei University College of Medicine, Seoul 03722, Korea; 2 Department of Microbiology and Immunology, Institute for Immunology and Immunological Diseases, Brain Korea 21 PLUS Project for Medical Science, Yonsei University College of Medicine, Seoul 03722, Korea; 3 College of Pharmacy, Seoul National University, Seoul 08826, Korea; 4 Division of Pediatric Neurology, Department of Pediatrics, Severance Children's Hospital, Epilepsy Research Institute, Seoul 03722, Korea

## Abstract

X-linked adrenoleukodystrophy (X-ALD), caused by an *ABCD1* mutation, is a progressive neurodegenerative disorder associated with the accumulation of very long-chain fatty acids (VLCFA). Cerebral inflammatory demyelination is the major feature of childhood cerebral ALD (CCALD), the most severe form of ALD, but its underlying mechanism remains poorly understood. Here, we identify the aberrant production of cholesterol 25-hydroxylase (*CH25H*) and 25-hydroxycholesterol (25-HC) in the cellular context of CCALD based on the analysis of ALD patient-derived induced pluripotent stem cells and *ex vivo* fibroblasts. Intriguingly, 25-HC, but not VLCFA, promotes robust NLRP3 inflammasome assembly and activation via potassium efflux-, mitochondrial reactive oxygen species (ROS)- and liver X receptor (LXR)-mediated pathways. Furthermore, stereotaxic injection of 25-HC into the corpus callosum of mouse brains induces microglial recruitment, interleukin-1β production, and oligodendrocyte cell death in an NLRP3 inflammasome-dependent manner. Collectively, our results indicate that 25-HC mediates the neuroinflammation of X-ALD via activation of the NLRP3 inflammasome.

## Abstract (web summary)

The mechanism underlying neuroinflammation in X-linked adrenoleukodystrophy (ALD) is poorly understood. Here authors identify aberrant production of 25-hydroxycholesterol (25-HC) in ALD patient-derived cells, and show that 25-HC mediates neuroinflammation via activating the NLRP3 inflammasome.

## Graphical abstract

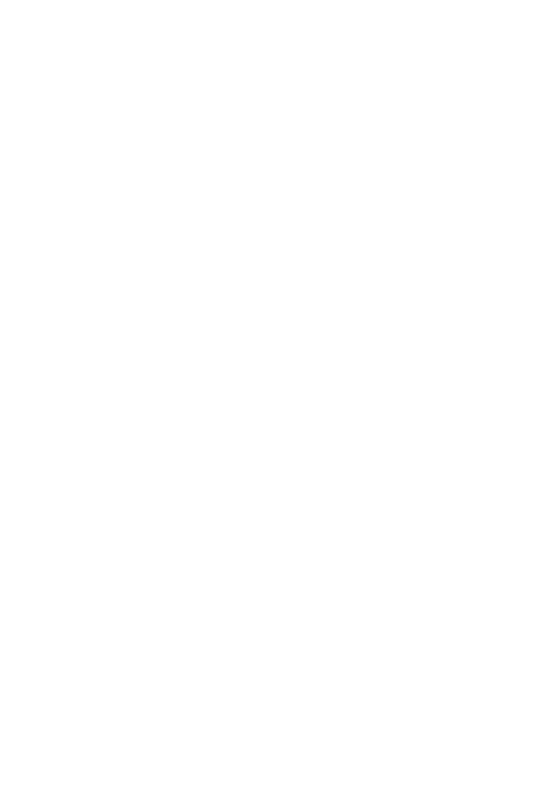

X-linked adrenoleukodystrophy (X-ALD) is an inherited metabolic disorder caused by a loss of function mutation in the ATP-binding cassette transporter subfamily D member 1 (*ABCD1*) gene^1^. ABCD1 mediates the transport of saturated very long-chain fatty acids (VLCFA) from the cytosol to the peroxisome for degradation^2^. Dysfunctional ABCD1, therefore, leads to the accumulation of VLCFA particularly in the adrenal cortex, central nervous system and plasma as well^3^,^4^. While abnormal accumulation of VLCFA is considered an important marker for X-ALD^5^,^6^, how VLCFA is involved in the pathogenesis of X-ALD remains to be clarified. The main manifestation of X-ALD is milder axonopathy of the spinal cord and more severe cerebral inflammatory demyelination^7^. The most severe phenotype of X-ALD is childhood cerebral ALD (CCALD), which is accompanied by acute inflammatory demyelination of the central nervous system that leads to a vegetative state or death within 3–5 years of onset^8^. On the other hand, adrenomyeloneuropathy (AMN), the most prevalent phenotype, manifests as a slowly progressive myelopathy in the adulthood^7^,^9^.

The presence of cerebral inflammation is a significant symptom of CCALD, but how cerebral inflammation is initiated or promoted in CCALD patients is still poorly understood. Nevertheless, abnormal accumulation of VLCFA is considered as a biochemical trigger for the pathophysiology of CCALD including cerebral inflammatory response^10^. Furthermore, the pathogenesis of cerebral ALD could be affected by a range of genetic and environmental factors^9^. Although *Abcd1*-deficient mice exhibited an increased level of VLCFA in the brain and adrenal gland, these mice did not show any characteristics of CCALD phenotypes including the cerebral pathology^11^. Thus, this lack of CCALD phenotype in *Abcd1*-deficient mice makes it more difficult to study the molecular basis of cerebral inflammation of CCALD caused by VLCFA accumulation.

A dysregulated innate immune response has been increasingly considered a potential contributor to cerebral inflammation and neurodegenerative disorders^12^. Of particular interest, nucleotide-binding oligomerization domain-like (NOD-like) receptor containing pyrin domain 3 (NLRP3) inflammasome signalling has been proposed as a crucial mediator in the progression of Alzheimer's diseases^13^,^14^. The inflammasome is a caspase-1-activating multi-protein complex that, once assembled, leads to the production of key pro-inflammatory cytokines interleukin-1β (IL-1β) and IL-18 (ref. 15). Unlike the other identified inflammasomes, NLRP3 can be activated by a wide range of stimulators from microbial toxin to host endogenous metabolites such as palmitate, cholesterol crystals and amyloid β (refs 15, 16). On the basis of its potent recognition capability under diverse stress conditions, NLRP3 is thought to be a crucial sensor of cellular abnormalities to initiate inflammation. The robust expression of the NLRP3 inflammasome components in mouse brain microglia was reported previously^17^. Of particular interest, *Nlrp3*-deficient mice exhibited delayed demyelination and oligodendrocyte depletion in a cuprizone-induced demyelination model^18^. In the present study, we used microarray analysis and identified 25-hydroxycholesterol (25-HC) as a potent mediator in the pathogenesis of X-ALD. Our data suggest that 25-HC, but not VLCFA, contributes to the cerebral inflammation of X-ALD via activation of the NLRP3 inflammasome pathway.

## Results

### CH25H upregulation in CCALD patient-derived cells

**Figure 1:**
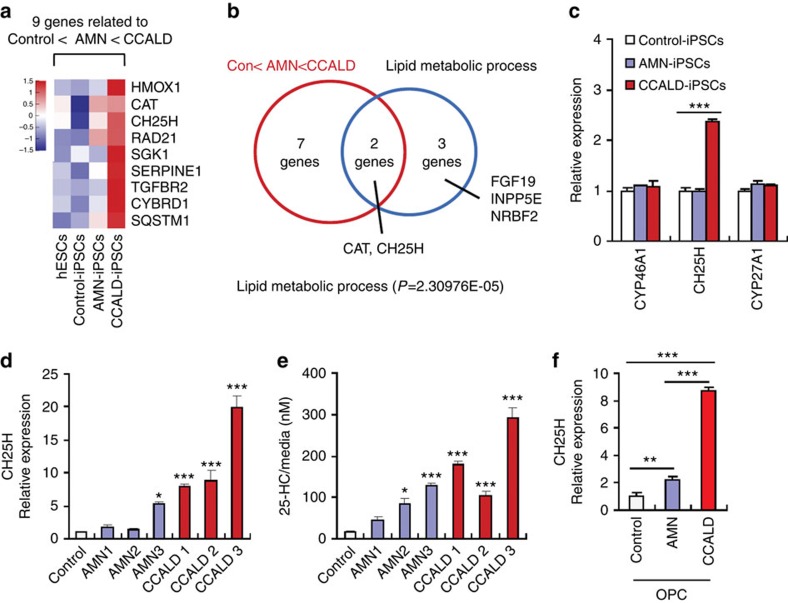
*CH25H* is upregulated in CCALD patient-derived cells. (**a**) Candidate genes significantly up- or downregulated (fold-change cutoff ≥2 and *P*<0.05) were selected based on the microarray analysis of human ESCs, control-, AMN- and CCALD-iPSCs. Heat map of nine selected genes showing the expression patterns correlated with disease severity via microarray analysis. (**b**) Venn diagram of overlapping genes between nine genes related to disease severity and five genes (among 70 genes) related to lipid metabolism. Catalase (*CAT*) and *CH25H* belong to both categories. (**c**) Relative mRNA expression of three cholesterol-hydroxylating enzymes in the control- and AMN- or CCALD-iPSCs measured by quantitative real-time PCR analysis. (*n*=6) (**d**,**e**) Levels of *CH25H* mRNA in the cell lysates (**d**) and of 25-HC in the culture supernatants (**e**) of control- (human dermal fibroblast), 3 AMN- and 3 CCALD-fibroblasts. (**d**, *n*=6; **e**, *n*=4) (**f**) Relative mRNA expression of *CH25H* in the control- or X-ALD-iPSC-derived oligodendrocyte precursor cells (AMN- or CCALD-OPCs). (*n*=6) For all panels, *P<0.05, **P<0.01 and ***P<0.001.

To recapitulate the distinctive features of X-ALD phenotypes, we have previously generated induced pluripotent stem cells (iPSCs) from the fibroblasts of healthy control and X-ALD patients including those with CCALD and AMN^19^. In early passage cultures of iPSCs, VLCFA levels were significantly higher in CCALD-iPSCs compared with control iPSCs, AMN-iPSCs or human embryonic stem cells (hESCs) (Supplementary Fig. 1). To gain molecular insight into the inflammatory phenotype of CCALD, we performed microarray-based transcriptional profiling analysis of hESCs, control-, AMN- and CCALD-iPSCs. We then identified 40 up- and 30 downregulated genes in the CCALD-iPSCs compared with control iPSCs (Supplementary Fig. 2). Among the 40 significantly upregulated genes, the expression level of nine genes was positively correlated with phenotypic severity in the following order (control<AMN<CCALD) (Fig. 1a). We also selected five candidate genes from 40 up- and 30 downregulated genes based on functional categorization of lipid metabolic processes for their potential implication in VLCFA accumulation. Venn diagram analysis indicated that *CH25H* (cholesterol 25-hydroxylase) and *CAT* (catalase) were simultaneously relevant to the phenotypic severity and lipid metabolism categories (Fig. 1b). CH25H is a hydroxylating enzyme that converts cholesterol to 25-hydroxycholesterol (25-HC)^20^, whereas CAT is a well-known antioxidant enzyme that catalyses the decomposition of hydrogen peroxide^21^. Given that oxysterols such as 25-HC are involved in the regulation of inflammation and also positively implicated in the pathogenesis of inflammatory or neurodegenerative diseases^22^,^23^,^24^, *CH25H* was then selected and examined for its potential role in the cerebral inflammatory phenotype of X-ALD.

In addition to CH25H, CYP46A1 (also known as cholesterol 24-hydroxylase) and CYP27A1 are other major oxysterol-producing enzymes that produce 24-HC and 27-HC, respectively^22^. However, the expression of *CYP46A1* or *CYP27A1* was not significantly altered in CCALD-iPSCs according to microarray analysis (Supplementary Table 1). Increased mRNA expression of *CH25H* was also validated in the CCALD-iPSCs by quantitative real-time PCR analysis, while the expression of *CYP46A1* and *CYP27A1* was similar in all three cell types (Fig. 1c).

To further validate the increased expression of *CH25H* of CCALD patients-derived cells, we compared the levels of *CH25H* in the *ex vivo* primary fibroblasts of healthy control, AMN patients and CCALD patients. Consistent with the expression pattern of iPSCs, all three CCALD patients' fibroblasts showed a significant increase in *CH25H* mRNA expression compared with control fibroblasts (Fig. 1d). In addition, significant upregulation of *CH25H* mRNA was observed in one AMN fibroblast, but not in two other AMN fibroblasts (Fig. 1d). Similarly, the level of 25-HC, a product of CH25H, also was significantly increased in the culture supernatants of all CCALD fibroblast cultures compared with control fibroblast cultures (Fig. 1e). To confirm the potential relevance of this finding in oligodendrocytes, which is the cell type primarily affected in the demyelination process of X-ALD, we generated oligodendrocyte precursor cells (CCALD-OPCs) differentiated from CCALD-iPSCs (Supplementary Fig. 3). Consistent with the above observations, *CH25H* mRNA levels were significantly higher in CCALD-OPCs than control- or AMN-OPCs (Fig. 1f). These findings strongly demonstrate that the expression of CH25H is significantly upregulated in CCALD-derived cells.

### ABCD1-dependent regulation of *CH25H* mRNA expression

**Figure 2:**
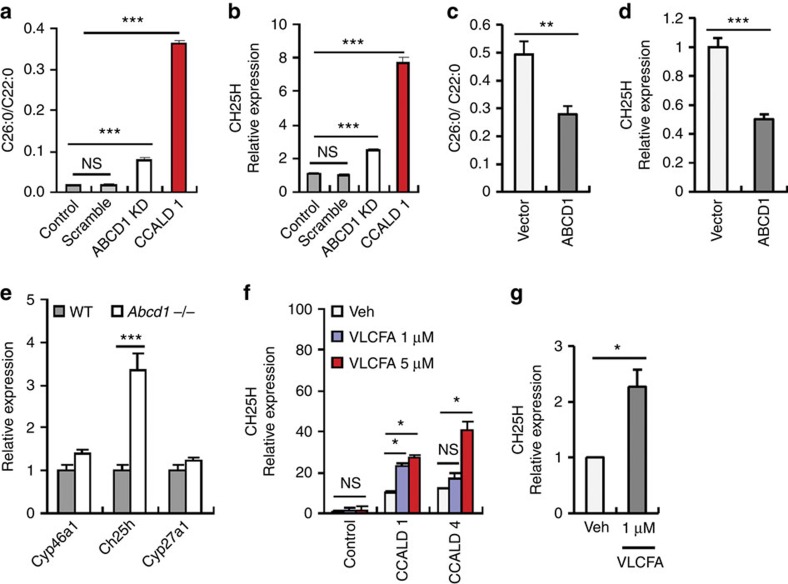
ABCD1 regulates the induction of *CH25H* expression in a VLCFA-dependent manner. (**a**,**b**) Cellular levels of VLCFA (**a**) and mRNA levels of *CH25H* (**b**) in control fibroblasts untransfected or transfected with scrambled- or *ABCD1*-targeting siRNA (ABCD1 KD) and in CCALD1 fibroblasts. (*n*=6) (**c**,**d**) Cellular levels of VLCFA (**c**) and mRNA levels of *CH25H* (**d**) in CCALD1 fibroblasts infected with lentiviral particles expressing empty vector or *ABCD1* gene. (*n*=5) (**e**) Relative mRNA expression of *Cyp46a1*, *Ch25h* and *Cyp27a1* in the brain homogenates of WT or *Abcd1*-deficient mice. (*n*=6). (**f**,**g**) Relative mRNA levels of *CH25H* in control- or two CCALD-fibroblasts (**f**) and in CCALD-OPCs (**g**) treated with vehicle (0.05% DMSO in PBS) or VLCFA at indicated concentration for 3 h. (**f**, *n*=6; **g**, *n*=5) For all panels, *P<0.05, **P<0.01, and ***P<0.001. NS, not significant.

To determine whether a defect in function of ABCD1 was involved in the induction of *CH25H* mRNA expression, we reduced *ABCD1* expression in control fibroblasts by siRNA-mediated knockdown (Supplementary Fig. 4a). Knockdown of *ABCD1* caused a significant increase in VLCFA levels and *CH25H* mRNA expression (Fig. 2a,b). Of note, CCALD-fibroblasts showed much higher levels of VLCFA and of *CH25H* mRNA than ABCD1-knockdowned fibroblasts (Fig. 2a,b). Conversely, ectopic expression of *ABCD1* in CCALD-fibroblasts significantly attenuated the level of VLCFA and *CH25H* mRNA (Supplementary Fig. 4b and Fig. 2c,d). These findings indicate that the dysfunction of *ABCD1* observed in X-ALD patients contributes to the induction of *CH25H* expression. Supporting these observations, increased *Ch25h* expression was also found in the brain homogenates of *Abcd1*-deficient mice compared with wild-type mice, while the expression of other cholesterol-metabolizing enzymes was unchanged (Fig. 2e).

Next, we examined whether VLCFA accumulation triggered by *ABCD1* dysfunction directly mediated the induction of *CH25H* expression. Exogenous addition of VLCFA did not affect the expression of *CH25H* in control fibroblasts, possibly due to the presence of a fully functional *ABCD1*, but led to significantly increased *CH25H* mRNA levels in CCALD-fibroblasts (Fig. 2f). Similarly, VLCFA treatment also led to increased expression of *CH25H* in CCALD-OPCs (Fig. 2g). These observations demonstrate that the accumulation of VLCFAs resulting from a defective *ABCD1* gene may account for the induction of *CH25H* expression in CCALD-derived cells.

### Microglial recruitment and IL-1β production by 25-HC

**Figure 3:**
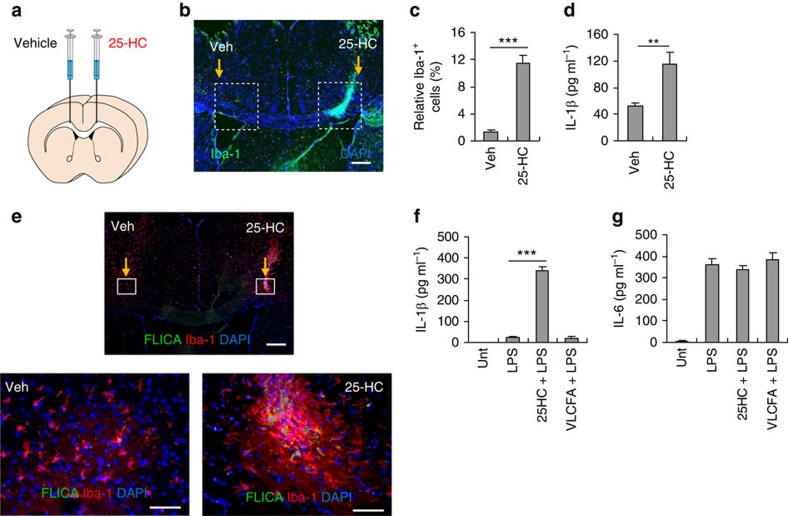
25-HC induces microglial recruitment and IL-1β production. (**a**) Illustration of stereotaxic injection of 25-HC (100 μM) or vehicle (0.1% ethanol in PBS) into the corpus callosum of mouse brain. (**b**) Representative immunofluorescence image of a coronal brain section stained with anti-Iba-1 antibody (green). DAPI represents the nuclear signal (blue). Scale bar, 200 μm. (**c**) Quantification of Iba-1-positive cells in the boxed areas of vehicle- or 25-HC-injected regions in **b**. (*n*=8) (**d**) Quantification of IL-1β in the brain homogenates of vehicle- or 25-HC-injected hemisphere. (*n*=11) (**e**) Representative immunofluorescence image of brain section co-stained with FLICA (green) and anti-Iba-1 antibody (red) after injection of vehicle or 25-HC. Magnified immunofluorescence images of the boxed areas in the upper panel are displayed in the lower panel: 25-HC-injected area (right) or vehicle-injected area (left). Scale bars, 200 μm (upper); or 50 μm (lower). (**f**,**g**) Quantification of IL-1β or IL-6 in the culture supernatants of mouse BMDMs untreated or treated with 25-HC (70 μM) or VLCFA (70 μM) for 10 h in the presence of LPS (0.25 μg ml^−1^, last 4 h) as indicated. (*n*=6). For all panels, **P<0.01 and ***P<0.001.

Recently, much attention has been paid to oxysterols, including 25-HC, as a potential regulator of innate or adaptive immune responses^23^,^24^,^25^. Given that neuroinflammation is closely related to the pathogenesis of CCALD^7^, we examined whether the elevated level of 25-HC could mediate cerebral inflammation. Therefore, we stereotactically injected 25-HC into the corpus callosum of wild-type mouse brains (Fig. 3a), and measured the recruitment of microglia, a major cell type for inducing neuroinflammation. To our surprise, significant recruitment of Iba-1-positive microglia was observed around the 25-HC-injected region, but not around the contralateral vehicle-injected region (Fig. 3b,c). Then, we examined whether 25-HC could mediate pro-inflammatory responses. Considering that pro-inflammatory cytokines such as TNF-α, IL-1β and IFN-γ were increased in the demyelination plaques of X-ALD brains^3^,^26^, these pro-inflammatory cytokines were measured in mouse brain homogenates. Among the tested cytokines, only IL-1β production was significantly increased in the 25-HC-injected hemisphere compared with the contralateral control region (Fig. 3d), whereas the levels of other pro-inflammatory cytokines such as TNF-α, IFN-γ, IL-6 and MCP-1 were unchanged in the two regions (Supplementary Fig. 5).

IL-1β is a key inflammatory cytokine that triggers pro-inflammatory responses at the site of inflamed tissues^27^. Unlike other cytokines, the production of functional IL-1β requires the additional post-translational regulator, caspase-1 (ref. 28). Thus, to validate whether the elevated level of IL-1β in the 25-HC-injected region resulted from the potentiated activation of caspase-1, we examined the presence of active caspase-1 in the corpus callosum of mouse brain using an active caspase-1-specific fluorochrome-labeled inhibitors of caspases (FLICA) probe, which yields green fluorescence in active-caspase-1-containing cells. Active caspase-1 was evident only in the microglia in the 25-HC-injected region (Fig. 3e), which indicated that the elevated IL-1β reflected the robust activation of caspase-1. Furthermore, an amoeboid morphology of the activated microglia was observed around the 25-HC-injected regions (Fig. 3e, lower right).

To further confirm whether 25-HC could trigger the production of IL-1β in cell culture, we examined the potential effect of 25-HC and VLCFA in mouse bone marrow-derived macrophages (BMDMs). Intriguingly, 25-HC, but not VLCFA, in the presence of lipopolysaccharide (LPS) co-treatment caused a marked secretion of IL-1β in BMDMs (Fig. 3f), whereas neither 25-HC nor VLCFA affected the LPS-stimulated secretion of IL-6 (Fig. 3g). However, 25-HC treatment did not induce mRNA expression of lL-1β and IL-6 in BMDMs (Supplementary Fig. 6a,b). These observations suggest that 25-HC, but not VLCFA, could activate the production of IL-1β in a transcription-independent manner.

### NLRP3 inflammasome assembly and activation by 25-HC

**Figure 4:**
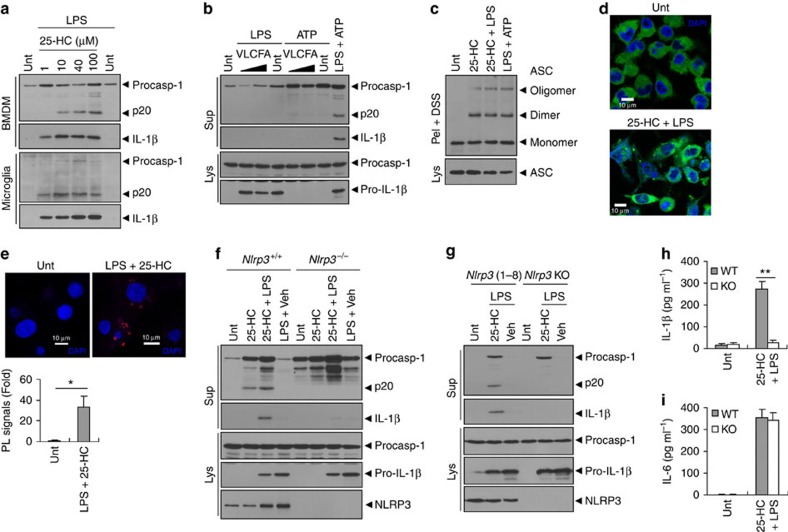
25-HC promotes the assembly and activation of the NLRP3 inflammasome. (**a**) Immunoblots of culture supernatants from mouse BMDMs and microglia untreated or treated with 25-HC (10 h) in the presence of LPS (last 4 h). (**b**) Immunoblots from BMDMs untreated or treated with VLCFA (40 or 100 μM, 10 h) in the presence of LPS (last 4 h) or ATP (2.5 mM, last 45 min), or primed with LPS (3 h), followed by ATP. (**c**) Immunoblots of DSS-crosslinked pellets (pel + DSS) or cellular lysates (Lys) from BMDMs untreated or treated with 25-HC in the presence of LPS, or treated with LPS, followed by ATP. (**d**) Confocal images of NLRP3-GFP-expressing macrophages untreated or treated with 25-HC (50 μM, 10 h) in the presence of LPS. DAPI represents the nuclear signal (blue). Scale bars, 10 μm. (**e**) Proximity-ligation (PL) assay of NLRP3 and ASC in BMDMs treated with 25-HC (50 μM, 10 h) in the presence of LPS. PL signals (red) represent the molecular association of NLRP3 and ASC. Data are shown as a representative image from five-independent samples (upper panel). DAPI represents the nuclear signal (blue). Scale bars, 10 μm. Relative intensity of PL signals (per DAPI signals) was determined and is displayed in the lower panel. (*n*=5) (**f**) *Nlrp3*^+/+^ or *Nlrp3*^−/−^ BMDMs were treated with 25-HC (100 μM) or vehicle (0.1% ethanol in PBS) in the presence of LPS (last 4 h) as indicated, and analysed by immunoblot. (**g**) *Nlrp3*-expressing (1–8) or *Nlrp3*-deficient immortalized BMDMs were treated with 25-HC in the presence of LPS as indicated and analysed by immunoblot. (**h**,**i**) Quantification of IL-1β (**h**) or IL-6 (**i**) in supernatant fractions of *Nlrp3*^+/+^ (WT) or *Nlrp3*^−/−^ (KO) microglial cells treated with 25-HC (50 μM) in the presence of LPS. (*n*=5) (**b**,**c**,**f**,**g**) Culture supernatants (Sup), cellular lysates (Lys) or DSS-crosslinked pellets (Pel + DSS) as indicated were analysed by immunoblot. For all panels, *P<0.05 and **P<0.01.

Activation of caspase-1 requires the assembly and activation of the inflammasome complex^15^. This led us to investigate whether heightened 25-HC production could activate inflammasome signalling. Consistent with the above results, 25-HC treatment promoted a robust processing of caspase-1 and secretion of active IL-1β in mouse BMDMs and microglia in the presence of LPS co-treatment (Fig. 4a). This 25-HC-triggered activation of caspase-1 and IL-1β was also found in mouse primary mixed glial cells and human THP-1 macrophages (Supplementary Fig. 7a,b). In contrast, VLCFA did not activate caspase-1 signalling in BMDMs (Fig. 4b), mixed glial cells, or THP-1 cells (Supplementary Fig. 8a,b), which demonstrated that caspase-1-activating capability was specific to 25-HC, but not to VLCFA. In particular, the microglia-enriched population exhibited a stronger activation of caspase-1 signalling than mixed glial cells containing <10% microglia (Supplementary Fig. 9a,b). Indeed, the expression of the major inflammasome components, apoptosis-associated speck-like protein containing a caspase-recruitment domain (ASC) and NLRP3, were increased in the microglia relative to mixed glial cells (Supplementary Fig. 9b). These data suggest that microglia might be the primary cell population that activates caspase-1 when 25-HC accumulates in the brain.

To provide molecular insight into the inflammasome activation by 25-HC, we next investigated ASC oligomerization, which is a critical step in inflammasome activation^29^. Consistent with the above data showing the secretion of active caspase-1 and IL-1β, 25-HC promoted a robust oligomerization of ASC as determined by a discuccinimidyl suberate (DSS)-crosslinking assay (Fig. 4c). Among the identified inflammasomes, NLRP3 shows the unique features of recognizing diverse endogenous danger signals such as palmitic acid^16^,^30^. To investigate the possibility that 25-HC could activate the NLRP3 inflammasome, we examined the oligomerization of NLRP3 using NLRP3-GFP-expressing macrophages. Noticeably, 25-HC treatment caused formation of speck-like aggregates of NLRP3 (Fig. 4d), indicating that 25-HC might act as another endogenous danger signal for NLRP3 activation. To further validate whether 25-HC could trigger the assembly of NLRP3 inflammasome, the spatial proximity of NLRP3 with ASC was measured using a proximity-ligation assay as recently described^31^. Consistent with the oligomerization of ASC and NLRP3, 25-HC treatment caused a marked increase in the proximity-ligation signal, which represented the molecular assembly of NLRP3 with ASC (Fig. 4e and Supplementary Fig. 10).

Supporting these observations, 25-HC-promoted caspase-1 processing and IL-1β secretion were completely abolished in *Nlrp3*-deficient BMDMs (Fig. 4f), microglia and mixed glial cells (Supplementary Fig. 11a,b). Conversely, the restoration of NLRP3 expression in *Nlrp3*^−/−^ immortalized BMDMs regained the susceptibility to 25-HC-induced caspase-1 activation (Fig. 4g). In correlation with these findings, 25-HC-triggered IL-1β secretion was largely impaired in *Nlrp3*-lacking microglia (Fig. 4h) and BMDMs (Supplementary Fig. 11c), while LPS-triggered IL-6 secretion was not affected by the absence of NLRP3 in either cell type (Fig. 4i and Supplementary Fig. 11d). These data strongly demonstrate that 25-HC is a potent inducer of the NLRP3 inflammasome assembly and activation.

### Molecular mechanism of NLRP3 activation by 25-HC

**Figure 5:**
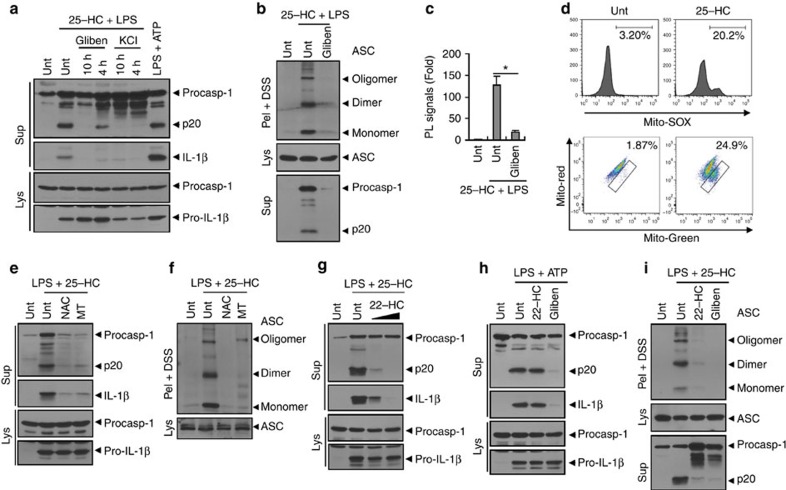
25-HC activates the NLRP3 inflammasome signalling in a potassium efflux/mitochondrial ROS/LXR-dependent manner. (**a**) Immunoblots of mouse BMDMs treated with 25-HC (50 μM, 10 h) and LPS (final 4 h) in the presence of glibenclamide (50 μM) or KCl (20 mM) for the indicated times, or treated with LPS, followed by ATP. (**b**) Immunoblots of BMDMs treated with 25-HC (50 μM, 10 h) and LPS (final 4 h) in the presence of glibenclamide (50 μM, 10 h). (**c**) PL assay of NLRP3 and ASC in microglia similarly treated as in **b**. Relative PL signals (per DAPI) are displayed. (*n*=5) *P<0.05 (**d**) Flow cytometric analysis of BMDMs treated with 25-HC (50 μM, 10 h) and LPS (final 3 h) after staining with MitoSOX (upper panel) or co-stained with MitoTracker Green and MitoTracker Deep Red (lower panel). (**e**,**f**) Immunoblots of BMDMs primed with LPS (3 h), followed by treatments with 25-HC (80 μM) in the presence of NAC (20 mM) or Mito-TEMPO (MT, 200 μM) for 6 h. (**g**,**h**) Immunoblots of BMDMs primed with LPS (3 h), followed by 25-HC (80 μM) in the presence of 22-HC (50 or 100 μM) for 6 h (**g**), or primed with LPS in the presence of 22-HC (100 μM) or glibenclamide (50 μM) for 3 h, followed by ATP (2 mM, 45 min) (**h**). (**i**) Immunoblots of BMDMs primed with LPS (3 h), followed by 25-HC (80 μM) in the presence of 22-HC (100 μM) or glibenclamide (50 μM) for 6 h. (**a**,**b**,**e**–**i**) Culture supernatants (Sup), cellular lysates (Lys) or DSS-crosslinked pellets (Pel + DSS) as indicated were analysed by immunoblot.

Despite the fact that diverse stimulators have been shown to activate NLRP3 inflammasomes, the detailed molecular mechanism of how NLRP3 is activated is not fully understood. To provide molecular insight into the NLRP3 activation by 25-HC, we first examined its dependence on potassium (K^+^) efflux, currently regarded as the most common mediator of NLRP3 activation in response to diverse stimulators^32^. The inhibition of K^+^ efflux by glibenclamide or high extracellular KCl markedly attenuated 25-HC-induced caspase-1 and IL-1β processing in mouse BMDMs (Fig. 5a). Consistently, glibenclamide also blocked the oligomerization of ASC (Fig. 5b) and the association of NLRP3 and ASC (Fig. 5c and Supplementary Fig. 12) in mouse BMDMs in response to 25-HC treatment. These findings indicate that K^+^ efflux is involved in the 25-HC-triggered activation of NLRP3 inflammasome.

In addition to K^+^ efflux, mitochondrial damage or mitochondrial ROS (mtROS) production is also considered a crucial mediator of NLRP3 activation in response to diverse NLRP3 agonists^16^,^33^. Interestingly, 25-HC treatment induced a considerable increase in mtROS-producing cells as measured using a mtROS-specific MitoSOX probe in BMDMs (Fig. 5d, upper panel). To provide more evidence that 25-HC could impair the function of mitochondria, we determined another indication of mitochondrial damage by co-staining with MitoTracker Green and MitoTracker Deep Red to measure the dissipation of mitochondrial membrane potential^33^,^34^,^35^. Consistent with mtROS production, treatment with 25-HC caused an obvious increase in the damaged mitochondria-containing cell population (Fig. 5d, lower panel). This increase in mtROS-producing or damaged mitochondria-containing cells was also observed in the *Nlrp3*-deficient BMDMs upon 25-HC stimulation (Supplementary Fig. 13a,b), indicating that these mitochondrial alterations by 25-HC were not consequences of caspase-1/inflammasome activation. Similarly, 25-HC treatment resulted in the production of mtROS in microglia (Supplementary Fig. 13a, lower panel). These observations indicate that 25-HC treatment could impair mitochondria in an inflammasome activity-independent manner.

Additionally, *N*-acetyl-cysteine (NAC), a general antioxidant, and Mito-TEMPO, a mitochondria-specific ROS scavenger, led to a marked reduction in caspase-1 activation (Fig. 5e) and ASC oligomerization (Fig. 5f) in BMDMs stimulated with 25-HC. As expected, Mito-TEMPO considerably attenuated the increase in mtROS-producing BMDMs (Supplementary Fig. 14a), suggesting that mtROS production might be related to the underlying mechanism of NLRP3 inflammasome actiation by 25-HC. Of note, the 25-HC-induced mitochondrial alterations were not an immediate, but rather a delayed phenomenon that was observed after at least 3 h of 25-HC treatment (Supplementary Fig. 14b). In correlation with this finding, 25-HC-induced caspase-1 activation was observed only after at least 6 h of 25-HC treatment (Supplementary Fig. 15a,b). On the basis of these data, we inferred that 25-HC caused the mitochondrial damage in the delayed response, which was likely associated with the NLRP3 activation by 25-HC.

A recent report showed that liver X receptor (LXR) ligands, such as TO901317 or 25-HC, promoted the caspase-1-dependent cell death of colon cancer cells^36^. Furthermore, the authors proposed that LXR ligands could induce the activation of NLRP3 inflammasome possibly through inducing the extracellular secretion of ATP^36^. Therefore, we examined the involvement of LXR signalling in the 25-HC-triggered activation of NLRP3 using a LXR antagonist, 22(S)-hydroxycholesterol (22(S)-HC). Co-treatment with 22(S)-HC showed a remarkable reduction of caspase-1 and IL-1β secretion in mouse BMDMs stimulated with 25-HC (Fig. 5g). However, 22(S)-HC did not abolish the ATP-stimulated canonical NLRP3 inflammasome activation (Fig. 5h). Consistently, 22(S)-HC also blocked the 25-HC-induced oligomerization of ASC (Fig. 5i). These data suggest that LXR signalling might be mediated in the 25-HC-triggered activation of NLRP3. In agreement with a recent report^36^, TO901317, another synthetic LXR agonist, exhibited the potent capability to activate caspase-1 (Supplementary Fig. 16a). Unlike 25-HC, TO901317-induced caspase-1 activation was observed in *Nlrp3*^−/−^ BMDMs (Supplementary Fig. 16a) and was not inhibited by the potassium efflux blocker glibenclamide (Supplementary Fig. 16b). These observations demonstrate that TO901317 could promote caspase-1 activation in an NLRP3-independent manner. Furthermore, TO901317 exhibited much higher toxicity to BMDMs compared with 25-HC (Supplementary Fig. 16c). On the basis of these findings, LXR signalling was potentially involved in NLRP3 inflammasome activation at least in the case of 25-HC stimulation. Supporting our data, the 25-HC-induced secretion of IL-1β was effectively suppressed by the K^+^ efflux blocker, the mtROS scavenger and the LXR antagonist (Supplementary Fig. 17).

### NLRP3 deficiency reduces cerebral inflammation by 25-HC

**Figure 6:**
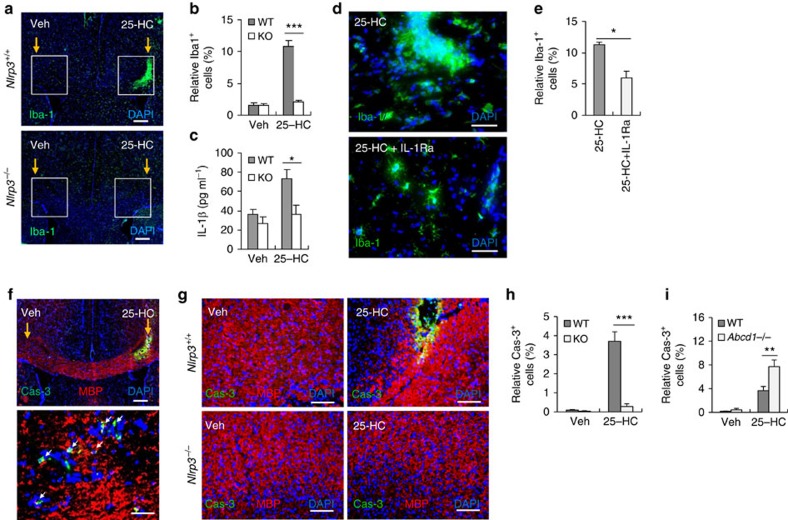
25-HC promotes the microglial recruitment and oligodendrocyte cell death in an NLRP3 inflammasome-dependent manner. (**a**) Immunohistochemical staining of coronal brain sections with anti-Iba-1 antibody (green) and DAPI (blue) after bilateral stereotaxic injection of 25-HC (100 μM) or vehicle into the brain of *Nlrp3*^+/+^ or *Nlrp3*^−/−^ mice. Scale bar, 200 μm. (**b**) Quantification of Iba-1^+^ microglia in confocal images of vehicle- or 25-HC-injected brains from *Nlrp3*^+/+^ (WT) or *Nlrp3*^−/−^ (KO) mice. (*n*=6). (**c**) Quantification of IL-1β in the brain homogenates of vehicle- or 25-HC-injected hemispheres from *Nlrp3*^+/+^ (WT) or *Nlrp3*^−/−^ (KO) mice. (*n*=7) (**d**) Immunohistochemical staining of brain sections with anti-Iba-1 antibody (green) after stereotaxic injection of 25-HC (100 μM) together with recombinant IL-1 receptor antagonist (IL-1Ra, 5 μg). Scale bar, 50 μm. (**e**) Quantification of Iba-1^+^ microglia in the confocal images of vehicle- or 25-HC-injected brain sections as treated in **d**. (*n*=4) (**f**) Immunohistochemical staining of brain sections with anti-active caspase-3 antibody (green), anti-myelin basic protein antibody (MBP, red), and DAPI (blue). Lower panel is representative magnified image of a 25-HC-injected region. Arrows indicate the active caspase-3-positive oligodendrocytes (yellow). Scale bars, 200 μm (upper); 50 μm (lower). (**g**) Immunohistochemical staining of brain sections of *Nlrp3*^+/+^ or *Nlrp3*^−/−^ mice stained as in **f** after stereotaxic injection of vehicle or 25-HC. Scale bar, 100 μm. (**h**) Quantification of active caspase-3^+^ oligodendrocytes in the confocal images of vehicle- or 25-HC-injected brain sections as treated in **g**. (*n*=6) (**i**) Quantification of active caspase-3^+^ oligodendrocytes in the confocal images of vehicle- or 25-HC-injected brain sections of *Abcd1*^+/+^ (WT) or *Abcd1*^−/−^ mice. (*n*=6) For all panels, *P<0.05, **P<0.01 and ***P<0.001.

To validate the contribution of the NLRP3 inflammasome to 25-HC-induced cerebral inflammation, we measured the recruitment of microglia after the stereotaxic injection of 25-HC into the corpus callosum of *Nlrp3*^+/+^ and *Nlrp3*^−/−^ mice. Recruitment of microglia into the 25-HC-injected region was significantly reduced in *Nlrp3*-deficient mice (Fig. 6a,b). Moreover, 25-HC-induced production of IL-1β was significantly attenuated in the brain of *Nlrp3*^−/−^ mice (Fig. 6c). To further support these data, we used a recombinant IL-1 receptor antagonist (IL-1Ra) to confirm whether microglial recruitment was inflammasome-dependent in our experiments. 25-HC-triggered recruitment of microglia was significantly reduced by the administration of the IL-1Ra (Fig. 6d,e), which indicated that IL-1β played a crucial role in the recruitment of microglia into the 25-HC-injected area.

Oligodendrocytes are the major myelin-forming cells of the central nervous system. 25-HC injection caused robust apoptotic cell death of oligodendrocytes in the 25-HC-injected region, but not in the vehicle-injected contralateral region (Fig. 6f). This finding suggested that the increased level of 25-HC was indeed responsible for the depletion of oligodendrocytes; it is possible that oligodendrocyte depletion could lead to the demyelination observed in X-ALD. To our surprise, this 25-HC-induced loss of oligodendrocytes were significantly attenuated in *Nlrp3*^−/−^ mice (Fig. 6g,h). These findings clearly demonstrate that 25-HC could cause oligodendrocytes apoptosis via activation of the NLRP3 inflammasome. In support of our data, *Abcd1*-deficient mice showed increased susceptibility to cerebral inflammations as shown by oligodendrocyte apoptosis (Fig. 6i and Supplementary Fig. 18a) and the recruitment of microglia (Supplementary Fig. 18b,c) in response to the stereotaxic administration of 25-HC. Our data collectively demonstrate that 25-HC promoted NLRP3 inflammasome assembly and activation that led to the recruitment of microglia and apoptosis of oligodendrocytes.

## Discussion

The underlying mechanism by which cerebral inflammation is initiated has been a long-standing conundrum in the pathogenesis of X-ALD^7^. VLCFA is considered the best biomarker of X-ALD^6^, but its contribution to the neuroinflammation of CCALD is still unclear^10^. In the present study, we identified the unique upregulation of *CH25H* and the subsequent production of 25-HC in CCALD-derived cells. Indeed, accumulated VLCFA was responsible for the induction of CH25H (Fig. 2f,g). *CH25H* was previously suggested as a risk factor for the pathogenesis of Alzheimer's diseases^37^,^38^, which indicated that *CH25H* induction and the subsequent production of 25-HC could contribute to the progression of neurodegenerative diseases. In correlation with this observation, our data showed that 25-HC could act as a potent mediator and inducer of cerebral inflammation in CCALD through its ability to activate the NLRP3 inflammasome.

25-HC was first identified as a feedback suppressor of cholesterol biosynthesis that acted mainly by inhibiting pathways involved with sterol response element-binding proteins^22^. *CH25H* is one of the highly inducible, interferon-stimulated genes expressed in response to viral infection^39^. Its product, 25-HC, affects multiple steps of the viral life cycle including the inhibition of viral entry, viral replication and particle assembly^23^,^39^. However, the contribution of 25-HC to the inflammatory response still remains to be further investigated, particularly in terms of transcriptional control of pro-inflammatory cytokine expressions. Previous literature reported that 25-HC could augment the inflammatory response, leading to increased secretion of pro-inflammatory cytokines, such as IL-1β and IL-6 (refs 40, 41, 42, 43). Recently, Gold *et al.* showed that *Ch25h*^−/−^ mice were less susceptible to influenza A virus infection due to a decreased inflammatory response, and proposed that 25-HC could amplify the production of toll-like receptor (TLR)-induced cytokines by facilitating AP-1-mediated transcription^40^. Supporting the pro-inflammatory role of 25-HC, another study demonstrated that 25-HC-triggered IL-1β production in co-cultures of human monocytes and vascular smooth muscle cells^41^. In correlation with its pro-inflammatory function, 25-HC has been considered to promote the progression of chronic inflammatory diseases, including atherosclerosis, chronic obstructive pulmonary disease, and Alzheimer's disease^37^,^44^,^45^. However, the precise role of 25-HC in inflammasome activation at the molecular level has been poorly described to date.

By contrast, a recent paper demonstrated increased secretion of IL-1β in *Ch25h*-deficient BMDMs upon LPS-ATP stimulation, and proposed that 25-HC could attenuate the production of pro-IL-1β by inhibiting sterol response element-binding proteins-mediated transcription^46^. We cannot fully explain these discrepancies about the contribution of 25-HC to inflammation at present. One possible explanation could be the different concentration of 25-HC used. For example, more than micromolar concentrations of 25-HC were employed in the studies proposing its pro-inflammatory action^40^,^41^,^42^,^43^, whereas nanomolar concentrations were used when documenting anti-inflammatory effects^46^. The physiological concentration of 25-HC in the brain is difficult to measure, but the level of 25-HC might possibly be elevated in the demyelinating plaque of CCALD due to the increased VLCFA level^47^,^48^. With these conflicting reports, we carefully examined the role of 25-HC in BMDMs, microglia, and human THP-1 cells, as well as in the mouse brain, and we provide definitive molecular evidences to support the pro-inflammatory role of 25-HC in NLRP3 inflammasome activation.

Like other classical NLRP3 agonists, 25-HC also induced a potassium efflux-dependent NLRP3 inflammasome activation. Emerging evidence has proposed a potential linkage between mitochondrial damage or damaged mitochondria-derived danger signals such as mtROS and activation of the NLRP3 inflammasome under diverse stress conditions^33^,^49^. Of note, 25-HC caused robust production of mtROS and mitochondrial damage, which was consistent with the previous report^50^. The scavenging of mtROS markedly attenuated 25-HC-induced caspase-1 activation and ASC oligomerization under our experimental conditions. Despite the poor understanding of how 25-HC causes mitochondrial dysfunction, mitochondrial damage and mtROS were shown to be closely related to the pathogenesis of ALD^51^,^52^,^53^. In this regard, it is plausible that mtROS production is involved in 25-HC-induced NLRP3 inflammasome activation that could possibly result in the pathophysiology of CCALD. In addition, our data showed that 25-HC alone without LPS co-treatment could activate NLRP3 inflammasome in BMDMs and microglia, but failed to induce IL-1β mRNA expression. Considering that NLRP3 inflammasome activation requires a priming step, it is questionable as to whether 25-HC could provide a priming signal for NLRP3 activation in the physiological context. Of interest, 25-HC alone promoted NLRP3-independent cell death of BMDMs and mixed glial cells (Supplementary Fig. 19), which suggested that 25-HC-triggered cell death could release a danger signal that functions as a priming signal under physiological conditions.

Given that 25-HC is a potent ligand of the LXR pathways^25^, we also investigated the involvement of LXR signalling in 25-HC-triggered NLRP3 inflammasome activation. A recent report consistently demonstrated that LXR ligands could promote the activation of NLRP3 inflammasome in colon cancer cells leading to cell death^36^. In our study, LXR signalling was likely to be involved in the 25-HC-induced caspase-1 activation of BMDMs. However, whether the engagement of LXR is a potent stimulator of NLRP3 remains to be elucidated. Another LXR agonist, TO901317, showed potent caspase-1 activation in BMDMs, as well as in *Nlrp3*-deficient cells. In this regard, further study will be required to address the precise role of the LXR pathways in inflammasome signalling.

The potential contribution of the NLRP3 inflammasome to chronic inflammatory disorders has been increasingly demonstrated^54^. In correlation with our study, the NLRP3 inflammasome was already shown to play a crucial role in demyelination and oligodendrocyte loss in a mouse model^18^, which suggested the significance of the NLRP3 inflammasome in cerebral neuroinflammation. Interestingly, endogenous molecules such as palmitate, uric acid crystals, cholesterol crystals and amyloid β, which are critical in the pathogenesis of chronic metabolic or neurodegenerative disorders, have been shown to activate the NLRP3 inflammasome^13^,^54^. Here, we present evidence that 25-HC could act as an endogenous NLRP3 stimulator leading to cerebral inflammation. Therefore, our data suggest that therapeutic interventions that can target the NLRP3 inflammasomes may show clinical benefit for the treatment of X-ALD.

## Methods

### Mice

C57BL/6, *Abcd1*^+/−^, and *Nlrp3*^−/−^ mice were obtained from The Jackson Laboratory and bred at Yonsei University College of medicine. All mice were on C57BL/6 background and 7–10 weeks male mice were used for the experiments. All mice were maintained under specific pathogen-free conditions. Protocols for the animal experiments were approved by the Institutional Ethical Committee, Yonsei University College of Medicine. All experiments were performed in accordance with the approved guidelines of the Institutional Ethical Committee.

### Establishment of X-ALD patients-derived cells

hESC and human iPSCs were maintained as described previously^19^. Human X-ALD-fibroblasts (GM04496 (CCALD1), GM04934 (CCALD 3), GM04932 (CCALD4), GM07675 (AMN 2), GM17819 (AMN3)) and control human dermal fibroblast (HDF, C-004-5C) cells were purchased from Coriell Institute and Invitrogen, respectively. Human fibroblasts were cultured as recommended by the manufacturer. Human X-ALD-fibroblasts were also derived and established from skin punch biopsies taken from a Korean CCALD patient (CCALD 2) and Korean AMN patient (AMN 1). The patients' clinical information is listed in the Supplementary Table 2. Human iPSCs were established from control- (HDF), AMN3- and CCALD1-fibroblasts. Differentiation of oligodendrocytes was performed as described previously^19^ and determined using anti-NG2 (Chemicon) and anti-A2B5 (Millipore) antibodies by flow cytometry.

### Ethical statement

Yonsei University Institutional Review Board approval (IRB 4-2016-0194) was obtained for the generation and analyses of fibroblasts from adrenoleukodystrophy patients. All volunteers who participated in this study signed written informed consent forms before skin biopsy sampling for human fibroblast establishment.

### Cell cultures

Mouse bone marrow cells were isolated from mouse femurs and differentiated into BMDMs with L929-conditioned medium^55^. Immortalized NLRP3-reconstituted BMDMs (N1-8) and NLRP3-GFP-expressing BMDMs were provided by E.S. Alnemri (Thomas Jefferson University, Philadelphia, PA, USA). All BMDMs were maintained in L929-conditioned DMEM supplemented with 10% FBS and antibiotics. THP-1 cells were differentiated into macrophage-like cells using a phorbol 12-myristate 13-acetate treatment and cultured in RPMI-1640 supplemented with 10% FBS, 2 mM glutamine, 10 mM HEPES, 1 mM sodium pyruvate, 0.05 mM 2-mercaptoethanol and antibiotics^56^. Mouse brain mixed glial cells were isolated from the whole brain of pups on the first postnatal day and cultured in DMEM/F-12 medium for 3 weeks as described previously^57^. Microglial cells were further enriched from the mixed glial cultures by mild trypsinization^58^. The microglia cultures were characterized by flow cytometry after co-staining with anti-CD11b and anti-F4/80 antibodies (eBioscience). Cell death was measured by the extracellular release of lactate dehydrogenase (LDH) using a CytoTox96 non-radioactive cytotoxicity assay kit (Promega).

### Microarray analysis

Microarray analysis was performed by Macrogen Inc. Briefly, 200 ng of total RNA from purified hESCs (P35–45), control iPSCs (P10–20), CCALD-iPSCs (P10–20), AMN-iPSCs (P10–20) was transcribed to yield biotinylated complementary RNA according to the manufacturer's instructions. Complementary RNA samples were each hybridized to a Human HT-12 v4.0 Expression Beadchip (Illumina). Arrays were scanned with an Illumina Bead Array Reader confocal scanner according to the manufacturer's instructions. Raw data were processed using the Illumina GenomeStudio v2011.1 (Gene Expression Module v1.9.0)). Gene-enrichment and functional annotation analysis for the significant probe list was performed using Gene Ontology (www.geneontology.org/). All data analysis and visualization of differentially expressed genes was conducted using R 3.1.2 (www.r-project.org). Microarray data have been deposited in the Gene Expression Omnibus database (GSE85804).

### Measurement of VLCFA and 25-HC

VLCFAs were measured as fatty acid methyl esters as described previously^19^. Briefly, after adding C27:0 as the internal standard, total lipid were extracted, converted to methyl esters, purified by thin-layer chromatography, and subjected to gas chromatography (GC). The level of 25-HC in culture media was determined by GC coupled with mass spectrometry (GC-MS) as described previously^59^. A deuterium-labelled internal standard (D6-25-HC) was used for calculating the concentration of 25-HC.

### ABCD1 expression

To knockdown *ABCD1* expression, cells were transfected with scrambled siRNA (4390843, Ambion) or ABCD1-targeting siRNAs (s1233, Invitrogen, 5′-CCUUCACUAUUGCCCGCAAtt-3′; 5′-UUGCGGGCAAUAGUGAAGGct-3′) using Lipofectamine RNAiMAX (Invitrogen) according to the manufacturer's instructions. To induce ectopic expression of *ABCD1*, cells were infected with lentiviral particles expressing vector or ABCD1. Lentiviral particles were prepared from the supernatants of 293GPG packaging cells transfected with empty or *ABCD1*-expressing lentiviral construct (Addgene).

### Quantification of mRNA

To measure mRNA production, total RNA was isolated using RNeasy Mini Kit (Intron) or Trizol reagent (Invitrogen) and reverse transcribed using Power cDNA synthesis kit (Intron). Quantitative real-time PCR were performed using SYBR Premix Ex Taq (Takara). Primers were as follows: (human *CYP46A1*) 5′-TGTGTTTTTGGATTG GGCTAAGA-3′ and 5′-ACTCAGGACTCGTGACGATGA-3′; (human *CH25H*) 5′-GCACTGCCTGCCAAGCGAGA-3′ and 5′-CTCCGCAGACAGCTCCCCCT-3′; (human *CYP27A1*) 5′-CGGCAACGGAGCTTAGAGG-3′ and 5′-GGCATAGCCTTGAACGAACAG-3′; (mouse *Cyp46a1*) 5′-GGACATCTCCCCTACTTTTGGA-3′ and 5′-GGACCATACTTCTTAGCCCAATC-3′; (mouse *Ch25h*) 5′-ATGGGCTGCTACAACGGTTC-3′ and 5′-CCTTGTCCTTATGGTGTCCCAG-3′; (mouse *Cyp27a1*) 5′-GCACAGGAGAGTACGGAGG-3′ and 5′-CGGGCAAGTGCAGCACATA-3′. Relative expression was expressed as means ± s.e.m. obtained from three independent experiments.

### Stereotaxic injection

Mice were anaesthetized with a mixture of tiletamine/zolazepam (Zoletil) and xylazine (Rompun) and mounted in a stereotaxic frame (Stoelting). The mice were injected with vehicle or 25-HC in the white matter of the corpus callosum over both hemispheres (1 or −1 mm lateral to the midline, 1 mm anterior to Bregma and 2 mm deep) via separate Hamilton syringes. In some experiments, recombinant human IL-1Ra (5 μg, Prospec) was co-injected with 25-HC. After 3 days, mice were anaesthetized and were transcardially perfused with paraformaldehyde. Brains were removed and were placed overnight at 4 °C in 4% (vol/vol) paraformaldehyde, then transferred to PBS. Coronal sections of 20 μm in thickness were prepared using a Vibratome microtome (Leica).

### Immunohistochemistry

Cells were fixed in 4% paraformaldehyde, permeabilized and stained with the primary antibodies targeting MBP (Abcam, ab7349, 1:100), cleaved caspase-3 (Cell Signaling Technology, 9661, 1:100) and Iba-1 (Wako, 019-19741, 1:100), followed by the appropriate Alexa Fluor 488- or 594-labelled secondary antibodies (Molecular Probes). DAPI counterstaining was used for nuclei visualization. Cell images were captured with an Olympus IX71 microscope and DP71 digital camera or Olympus FSX100 system. Iba-1-positive cells in the selected regions were counted using Image J.

### Immunoblot analysis

Cells were lysed in buffer containing 20 mM HEPES (pH 7.5), 0.5% Nonidet P-40, 50 mM KCl, 150 mM NaCl, 1.5 mM MgCl_2_, 1 mM EGTA and protease inhibitors. Soluble lysates were fractionated by SDS–polyacrylamide gel electrophoresis and then transferred to polyvinylidene difluoride membranes. In some experiments, cell culture supernatants were precipitated by the addition of methanol/chloroform mixture as described previously^60^ and then immunoblotted. The following antibodies were used for detecting mouse caspase-1 (Adipogen AG-20B-0042, 1:2,000), mouse IL-1β (R&D AF-401-NA, 1:1,000), human caspase-1 (Santa Cruz SC-515, 1:500), human IL-1β (Cell Signaling 2022, 1:1,000), mouse NLRP3 (Adipogen AG-20B-0014, 1:2,000) and mouse ASC (Santa Cruz SC-22514-R, 1:500). All the blots shown are representative image of at least three independent experiments. Images have been cropped for presentation. Full size images are presented in Supplementary Fig. 20.

### Cytokine production assay

The levels of IL-1β or IL-6 in the culture supernatants or the mouse brain homogenates were quantified by mouse IL-1β or IL-6 ELISA (R&D). For the brain homogenates, inflammatory cytokines were also quantified using the Cytometric Bead Array (CBA) Mouse Inflammatory Kit (BD). All the assay experiments were performed according to the manufacturer's protocols.

### Assay of inflammasome/caspase-1 activation

To induce a conventional NLRP3 inflammasome activation, BMDMs or microglial cells were primed with LPS (0.25 μg ml^−1^, 3 h), followed with ATP treatment (2∼2.5 mM, 45 min). To measure the inflammasome-activating role of 25-HC or VLCFA, cells were treated with 25-HC (1∼100 μM) or VLCFA (up to 100 μM) for 10 h in the presence of LPS treatment (0.25 μg ml^−1^, last 4 h). In some experiments, cells were first primed with LPS (0.25 μg ml^−1^, 3 h), washed with PBS, and then treated with 25-HC or other agonists as indicated. Inflammasome activation was determined by the presence of active caspase-1 p20 (mouse BMDM) or p10 (human THP-1 cells), and active IL-1β from culture supernatants in immunoblots, and by the extracellular IL-1β quantification using ELISA. FAM-FLICA-caspase-1 assay kit (ImmunoChemistry) was employed to detect active caspase-1 in the brain sections according to the manufacturer's protocol.

### Assay of NLRP3 inflammasome assembly

To measure the oligomerization of NLRP3, confocal microscopy of NLRP3-GFP-expressing BMDMs was performed as described previously^61^. To quantify NLRP3 speck formation, the relative percentage of NLRP speck-containing cells was determined by dividing the number of NLRP3 speck-containing cells by the number of total cells. To determine the oligomerization of ASC, DSS-mediated crosslinking assay was performed as described previously^62^. To visualize the molecular interaction of NLRP3 with ASC, proximity-ligation assay was performed using Duolink *In Situ* Red starter kit (Sigma) using anti-ASC or anti-NLRP3 antibodies according to the manufacturer's protocols. The relative proximity-ligation signals (PL signals/DAPI signals) were quantified using Image J and calculated as a relative fold-change compared with untreated controls.

### Measurement of mitochondrial ROS and damage

To measure mROS production, cells were stained with MitoSOX (Invitrogen) according to the manufacturer's protocol and the fluorescence of the cells was then monitored and analysed by flow cytometry (FACSVerse, BD). To assess mitochondrial membrane potential-dependent damage, cells were co-stained with MitoTracker Deep Red and MitoTracker Green (Invitrogen) according to the manufacturer's protocol and analysed by flow cytometry. All flow cytometry data are representative of at least three independent experiments.

### Statistical analysis

All values are expressed as the mean±s.e.m. of individual samples or independent experiments. Data were analysed using the Student's *t* test, and *P* values≤0.05 were considered significant.

### Data availability

Microarray data that support the findings of this study have been deposited in the Gene Expression Omnibus (GEO) database with the accession code GSE85804.

## Additional information

**How to cite this article:** Jang, J. *et al.* 25-hydroxycholesterol contributes to cerebral inflammation of X-linked adrenoleukodystrophy through activation of the NLRP3 inflammasome. *Nat. Commun.* **7**, 13129 doi: 10.1038/ncomms13129 (2016).

## Supplementary Material

Supplementary Information Supplementary Figures 1-20 and Supplementary Tables 1-2.

## References

[b1] Mosser J. *et al.* Putative X-linked adrenoleukodystrophy gene shares unexpected homology with ABC transporters. Nature 361, 726–730 (1993). 8441467 10.1038/361726a0

[b2] Singh I., Moser A. E., Goldfischer S. & Moser H. W. Lignoceric acid is oxidized in the peroxisome: implications for the Zellweger cerebro-hepato-renal syndrome and adrenoleukodystrophy. Proc. Natl Acad. Sci. USA 81, 4203–4207 (1984). 6588384 10.1073/pnas.81.13.4203 PMC345397

[b3] Powers J. M., Liu Y., Moser A. B. & Moser H. W. The inflammatory myelinopathy of adreno-leukodystrophy: cells, effector molecules, and pathogenetic implications. J. Neuropathol. Exp. Neurol. 51, 630–643 (1992). 1362438 10.1097/00005072-199211000-00007

[b4] van de Beek M. C. *et al.* C26:0-carnitine is a new biomarker for X-linked adrenoleukodystrophy in mice and man. PLoS ONE 11, e0154597 (2016). 27124591 10.1371/journal.pone.0154597 PMC4849772

[b5] Moser H. W. *et al.* Adrenoleukodystrophy: increased plasma content of saturated very long chain fatty acids. Neurology 31, 1241–1249 (1981). 7202134 10.1212/wnl.31.10.1241

[b6] Moser A. B. *et al.* Plasma very long chain fatty acids in 3,000 peroxisome disease patients and 29,000 controls. Ann. Neurol. 45, 100–110 (1999). 9894883 10.1002/1531-8249(199901)45:1<100::aid-art16>3.0.co;2-u

[b7] Berger J., Forss-Petter S. & Eichler F. S. Pathophysiology of X-linked adrenoleukodystrophy. Biochimie 98, 135–142 (2014). 24316281 10.1016/j.biochi.2013.11.023 PMC3988840

[b8] Moser H. W., Mahmood A. & Raymond G. V. X-linked adrenoleukodystrophy. Nat. Clin. Pract. Neurol. 3, 140–151 (2007). 17342190 10.1038/ncpneuro0421

[b9] Kemp S., Huffnagel I. C., Linthorst G. E., Wanders R. J. & Engelen M. Adrenoleukodystrophy—neuroendocrine pathogenesis and redefinition of natural history. Nat. Rev. Endocrinol 12, 606–615 (2016). 27312864 10.1038/nrendo.2016.90

[b10] Eichler F. S. *et al.* Is microglial apoptosis an early pathogenic change in cerebral X-linked adrenoleukodystrophy? Ann. Neurol. 63, 729–742 (2008). 18571777 10.1002/ana.21391

[b11] Lu J. F. *et al.* A mouse model for X-linked adrenoleukodystrophy. Proc. Natl Acad. Sci. USA 94, 9366–9371 (1997). 9256488 10.1073/pnas.94.17.9366 PMC23196

[b12] Heneka M. T., Kummer M. P. & Latz E. Innate immune activation in neurodegenerative disease. Nat. Rev. Immunol. 14, 463–477 (2014). 24962261 10.1038/nri3705

[b13] Halle A. *et al.* The NALP3 inflammasome is involved in the innate immune response to amyloid-beta. Nat. Immunol. 9, 857–865 (2008). 18604209 10.1038/ni.1636 PMC3101478

[b14] Heneka M. T. *et al.* NLRP3 is activated in Alzheimer's disease and contributes to pathology in APP/PS1 mice. Nature 493, 674–678 (2013). 23254930 10.1038/nature11729 PMC3812809

[b15] Schroder K. & Tschopp J. The inflammasomes. Cell 140, 821–832 (2010). 20303873 10.1016/j.cell.2010.01.040

[b16] Elliott E. I. & Sutterwala F. S. Initiation and perpetuation of NLRP3 inflammasome activation and assembly. Immunol. Rev. 265, 35–52 (2015). 25879282 10.1111/imr.12286 PMC4400874

[b17] Gustin A. *et al.* NLRP3 inflammasome is expressed and functional in mouse brain microglia but not in astrocytes. PLoS ONE 10, e0130624 (2015). 26091541 10.1371/journal.pone.0130624 PMC4474809

[b18] Jha S. *et al.* The inflammasome sensor, NLRP3, regulates CNS inflammation and demyelination via caspase-1 and interleukin-18. J. Neurosci. 30, 15811–15820 (2010). 21106820 10.1523/JNEUROSCI.4088-10.2010 PMC6633756

[b19] Jang J. *et al.* Induced pluripotent stem cell models from X-linked adrenoleukodystrophy patients. Ann. Neurol. 70, 402–409 (2011). 21721033 10.1002/ana.22486

[b20] Lund E. G., Kerr T. A., Sakai J., Li W. P. & Russell D. W. cDNA cloning of mouse and human cholesterol 25-hydroxylases, polytopic membrane proteins that synthesize a potent oxysterol regulator of lipid metabolism. J. Biol. Chem. 273, 34316–34327 (1998). 9852097 10.1074/jbc.273.51.34316

[b21] Quan F., Korneluk R. G., Tropak M. B. & Gravel R. A. Isolation and characterization of the human catalase gene. Nucleic Acids Res. 14, 5321–5335 (1986). 3755525 10.1093/nar/14.13.5321 PMC311543

[b22] Russell D. W. Oxysterol biosynthetic enzymes. Biochim. Biophys. Acta 1529, 126–135 (2000). 11111082 10.1016/s1388-1981(00)00142-6

[b23] Cyster J. G., Dang E. V., Reboldi A. & Yi T. 25-Hydroxycholesterols in innate and adaptive immunity. Nat. Rev. Immunol. 14, 731–743 (2014). 25324126 10.1038/nri3755

[b24] Poli G., Biasi F. & Leonarduzzi G. Oxysterols in the pathogenesis of major chronic diseases. Redox Biol 1, 125–130 (2013). 24024145 10.1016/j.redox.2012.12.001 PMC3757713

[b25] Tall A. R. & Yvan-Charvet L. Cholesterol, inflammation and innate immunity. Nat. Rev. Immunol. 15, 104–116 (2015). 25614320 10.1038/nri3793 PMC4669071

[b26] McGuinness M. C. *et al.* Tumor necrosis factor-alpha and X-linked adrenoleukodystrophy. J. Neuroimmunol. 61, 161–169 (1995). 7593551 10.1016/0165-5728(95)00084-f

[b27] Fantuzzi G. & Dinarello C. A. The inflammatory response in interleukin-1 beta-deficient mice: comparison with other cytokine-related knock-out mice. J. Leukoc. Biol. 59, 489–493 (1996). 8613694 10.1002/jlb.59.4.489

[b28] Kostura M. J. *et al.* Identification of a monocyte specific pre-interleukin 1 beta convertase activity. Proc. Natl Acad. Sci. USA 86, 5227–5231 (1989). 2787508 10.1073/pnas.86.14.5227 PMC297594

[b29] Fernandes-Alnemri T. *et al.* The pyroptosome: a supramolecular assembly of ASC dimers mediating inflammatory cell death via caspase-1 activation. Cell Death Differ. 14, 1590–1604 (2007). 17599095 10.1038/sj.cdd.4402194 PMC3345951

[b30] Wen H. *et al.* Fatty acid-induced NLRP3-ASC inflammasome activation interferes with insulin signaling. Nat. Immunol. 12, 408–415 (2011). 21478880 10.1038/ni.2022 PMC4090391

[b31] Misawa T. *et al.* Microtubule-driven spatial arrangement of mitochondria promotes activation of the NLRP3 inflammasome. Nat. Immunol. 14, 454–460 (2013). 23502856 10.1038/ni.2550

[b32] Munoz-Planillo R. *et al.* K^+^ efflux is the common trigger of NLRP3 inflammasome activation by bacterial toxins and particulate matter. Immunity 38, 1142–1153 (2013). 23809161 10.1016/j.immuni.2013.05.016 PMC3730833

[b33] Zhou R., Yazdi A. S., Menu P. & Tschopp J. A role for mitochondria in NLRP3 inflammasome activation. Nature 469, 221–225 (2011). 21124315 10.1038/nature09663

[b34] Won J. H., Park S., Hong S., Son S. & Yu J. W. Rotenone-induced impairment of mitochondrial electron transport chain confers a selective priming signal for NLRP3 inflammasome activation. J. Biol. Chem. 290, 27425–27437 (2015). 26416893 10.1074/jbc.M115.667063 PMC4646374

[b35] Park S. *et al.* Defective mitochondrial fission augments NLRP3 inflammasome activation. Sci. Rep. 5, 15489 (2015). 26489382 10.1038/srep15489 PMC4614538

[b36] Derangere V. *et al.* Liver X receptor beta activation induces pyroptosis of human and murine colon cancer cells. Cell Death Differ. 21, 1914–1924 (2014). 25124554 10.1038/cdd.2014.117 PMC4227150

[b37] Papassotiropoulos A. *et al.* Cholesterol 25-hydroxylase on chromosome 10q is a susceptibility gene for sporadic Alzheimer's disease. Neurodegener. Dis. 2, 233–241 (2005). 16909003 10.1159/000090362

[b38] Shibata N. *et al.* Association studies of cholesterol metabolism genes (CH25H, ABCA1 and CH24H) in Alzheimer's disease. Neurosci. Lett. 391, 142–146 (2006). 16157450 10.1016/j.neulet.2005.08.048

[b39] Blanc M. *et al.* The transcription factor STAT-1 couples macrophage synthesis of 25-hydroxycholesterol to the interferon antiviral response. Immunity 38, 106–118 (2013). 23273843 10.1016/j.immuni.2012.11.004 PMC3556782

[b40] Gold E. S. *et al.* 25-Hydroxycholesterol acts as an amplifier of inflammatory signaling. Proc. Natl Acad. Sci. USA 111, 10666–10671 (2014). 24994901 10.1073/pnas.1404271111 PMC4115544

[b41] Fu H. *et al.* Interleukin-1 potently contributes to 25-hydroxycholesterol-induced synergistic cytokine production in smooth muscle cell-monocyte interactions. Atherosclerosis 237, 443–452 (2014). 25463072 10.1016/j.atherosclerosis.2014.10.002

[b42] Rosklint T., Ohlsson B. G., Wiklund O., Noren K. & Hulten L. M. Oxysterols induce interleukin-1beta production in human macrophages. Eur. J. Clin. Invest. 32, 35–42 (2002). 10.1046/j.1365-2362.2002.00931.x 11851725

[b43] Koarai A. *et al.* 25-Hydroxycholesterol enhances cytokine release and Toll-like receptor 3 response in airway epithelial cells. Respir. Res. 13, 63 (2012). 22849850 10.1186/1465-9921-13-63 PMC3460764

[b44] Brown A. J. & Jessup W. Oxysterols and atherosclerosis. Atherosclerosis 142, 1–28 (1999). 9920502 10.1016/s0021-9150(98)00196-8

[b45] Sugiura H. *et al.* Increased 25-hydroxycholesterol concentrations in the lungs of patients with chronic obstructive pulmonary disease. Respirology 17, 533–540 (2012). 22295989 10.1111/j.1440-1843.2012.02136.x

[b46] Reboldi A. *et al.* Inflammation. 25-Hydroxycholesterol suppresses interleukin-1-driven inflammation downstream of type I interferon. Science 345, 679–684 (2014). 25104388 10.1126/science.1254790 PMC4289637

[b47] Paintlia A. S. *et al.* Correlation of very long chain fatty acid accumulation and inflammatory disease progression in childhood X-ALD: implications for potential therapies. Neurobiol. Dis. 14, 425–439 (2003). 14678759 10.1016/j.nbd.2003.08.013

[b48] Theda C., Moser A. B., Powers J. M. & Moser H. W. Phospholipids in X-linked adrenoleukodystrophy white matter: fatty acid abnormalities before the onset of demyelination. J. Neurol. Sci. 110, 195–204 (1992). 1506859 10.1016/0022-510x(92)90028-j

[b49] Nakahira K. *et al.* Autophagy proteins regulate innate immune responses by inhibiting the release of mitochondrial DNA mediated by the NALP3 inflammasome. Nat. Immunol. 12, 222–230 (2011). 21151103 10.1038/ni.1980 PMC3079381

[b50] Choi Y. K., Kim Y. S., Choi I. Y., Kim S. W. & Kim W. K. 25-hydroxycholesterol induces mitochondria-dependent apoptosis via activation of glycogen synthase kinase-3beta in PC12 cells. Free Radic. Res. 42, 544–553 (2008). 18569012 10.1080/10715760802146062

[b51] Lopez-Erauskin J. *et al.* Antioxidants halt axonal degeneration in a mouse model of X-adrenoleukodystrophy. Ann. Neurol. 70, 84–92 (2011). 21786300 10.1002/ana.22363 PMC3229843

[b52] Lopez-Erauskin J. *et al.* Impaired mitochondrial oxidative phosphorylation in the peroxisomal disease X-linked adrenoleukodystrophy. Hum. Mol. Genet 22, 3296–3305 (2013). 23604518 10.1093/hmg/ddt186

[b53] Kartha R. V. *et al.* Mechanisms of antioxidant induction with high-dose *N*-acetylcysteine in childhood cerebral adrenoleukodystrophy. CNS Drugs 29, 1041–1047 (2015). 26670322 10.1007/s40263-015-0300-9

[b54] Henao-Mejia J., Elinav E., Thaiss C. A. & Flavell R. A. Inflammasomes and metabolic disease. Annu. Rev. Physiol. 76, 57–78 (2014). 24274736 10.1146/annurev-physiol-021113-170324

[b55] Fernandes-Alnemri T. *et al.* The AIM2 inflammasome is critical for innate immunity to *Francisella tularensis*. Nat. Immunol. 11, 385–393 (2010). 20351693 10.1038/ni.1859 PMC3111085

[b56] Hwang I. *et al.* Non-transcriptional regulation of NLRP3 inflammasome signaling by IL-4. Immunol. Cell Biol. 93, 591–599 (2015). 25601272 10.1038/icb.2014.125 PMC4496256

[b57] Kim E. H., Won J. H., Hwang I. & Yu J. W. Cobalt chloride-induced hypoxia ameliorates NLRP3-mediated caspase-1 activation in mixed glial cultures. Immune Netw. 13, 141–147 (2013). 24009541 10.4110/in.2013.13.4.141 PMC3759711

[b58] Saura J., Tusell J. M. & Serratosa J. High-yield isolation of murine microglia by mild trypsinization. Glia 44, 183–189 (2003). 14603460 10.1002/glia.10274

[b59] Dzeletovic S., Breuer O., Lund E. & Diczfalusy U. Determination of cholesterol oxidation products in human plasma by isotope dilution-mass spectrometry. Anal. Biochem. 225, 73–80 (1995). 7778789 10.1006/abio.1995.1110

[b60] Fernandes-Alnemri T., Yu J. W., Datta P., Wu J. & Alnemri E. S. AIM2 activates the inflammasome and cell death in response to cytoplasmic DNA. Nature 458, 509–513 (2009). 19158676 10.1038/nature07710 PMC2862225

[b61] Park S. *et al.* The mitochondrial antiviral protein MAVS associates with NLRP3 and regulates its inflammasome activity. J. Immunol. 191, 4358–4366 (2013). 24048902 10.4049/jimmunol.1301170 PMC3848201

[b62] Yu J. W. *et al.* Pyrin activates the ASC pyroptosome in response to engagement by autoinflammatory PSTPIP1 mutants. Mol. Cell 28, 214–227 (2007). 17964261 10.1016/j.molcel.2007.08.029 PMC2719761

