# Supplementary material for: 25-hydroxycholesterol contributes to cerebral inflammation of X-linked adrenoleukodystrophy through activation of the NLRP3 inflammasome
Source: Nat Commun. 2016 Oct 25;7:13129. doi: 10.1038/ncomms13129 (PMC5093305; doi:10.1038/ncomms13129)
Supplement: Supplementary Information — Supplementary Figures 1-20 and Supplementary Tables 1-2. [file ncomms13129-s1.pdf]

Supplementary Information:

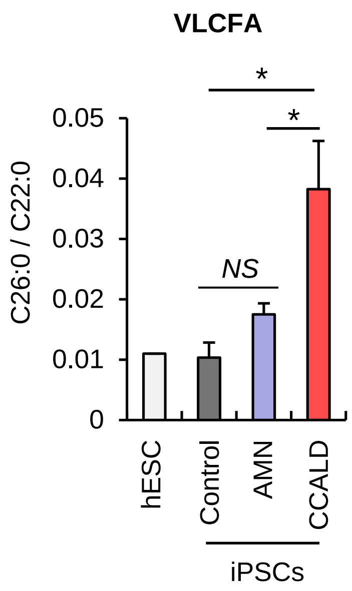

Supplementary Figure 1. Elevated level of VLCFA in the CCALD-iPSCs. VLCFA levels in human embryonic stem cells (hESCs) and iPSCs derived from the fibroblasts of controls, AMN patients, or CCALD patients. $*P<0.05$ ($n = 4$). NS, not significant.

Microarray comparison
in hESCs, control-iPSCs, AMN-iPSCs, and CCALD-iPSCs

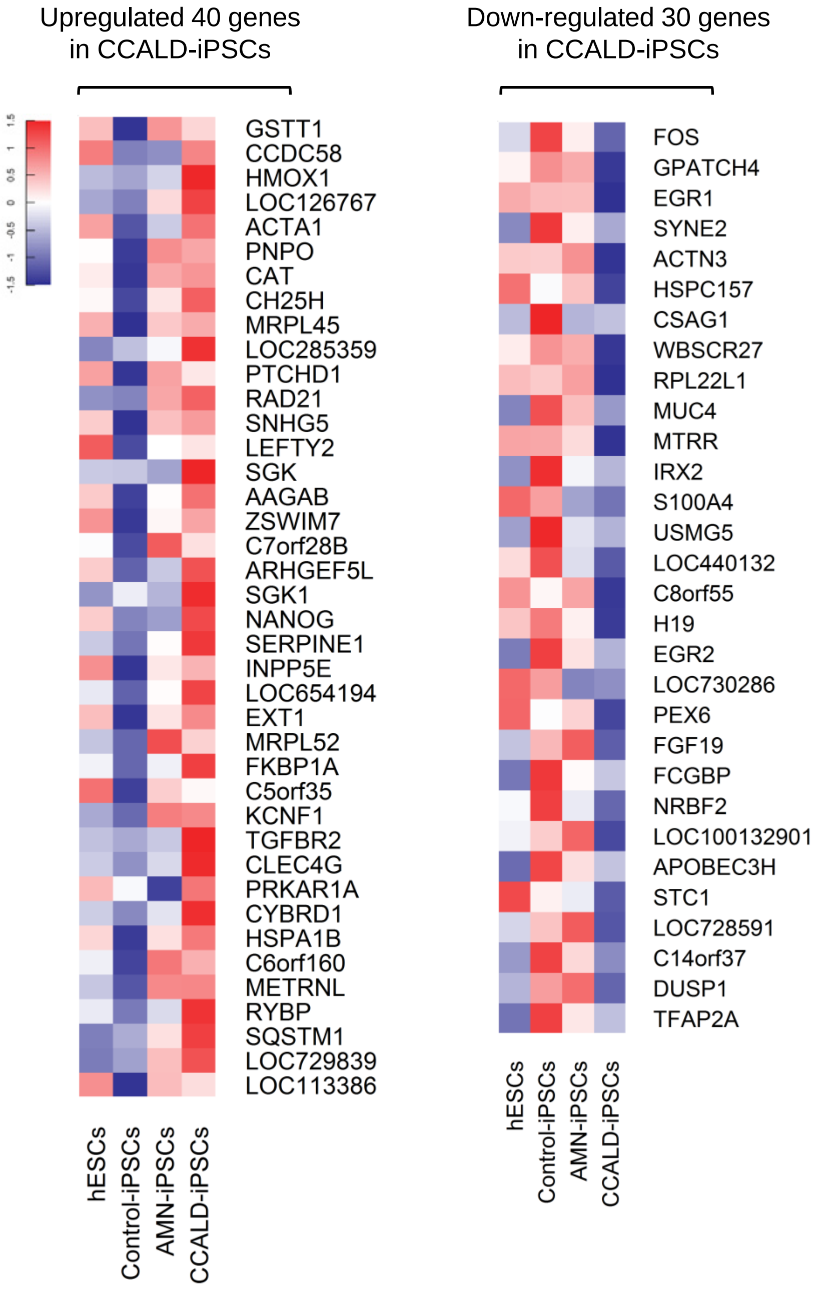

Supplementary Figure 2. Microarray analysis of control-iPSCs and X-ALD-iPSCs. Based on microarray analysis, up-regulated or down-regulated genes showing more than two-fold changes in CCALD-iPSCs compared with control-iPSCs were selected. Heatmap of up-regulated (left) and down-regulated (right) candidate genes in CCALD-iPSCs. Red and blue colors indicate up- or down-regulation, respectively.

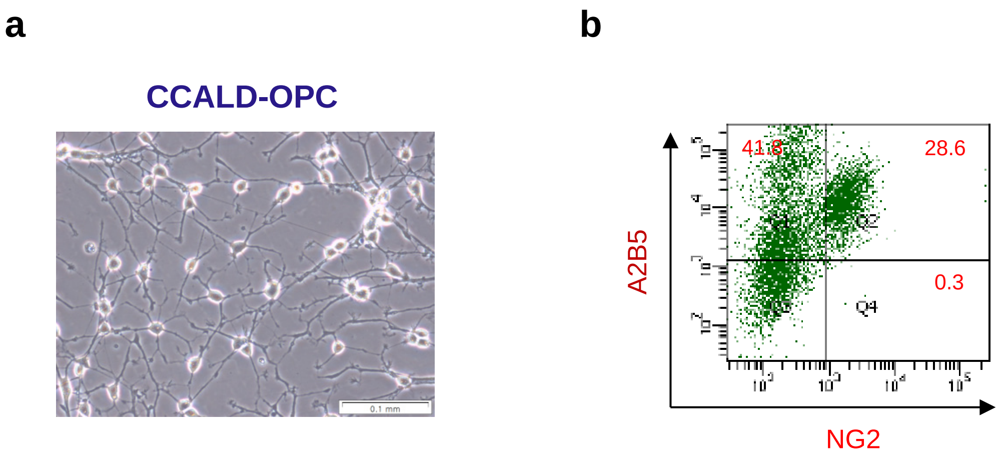

Supplementary Figure 3. Generation of CCALD-iPSCs-derived oligodendrocyte precursor cells (CCALD-OPC). (**a**) A representative phase contrast image of CCALD-OPC. Scale bars, 20 μm. (**b**) Representative fluorescence-activated cell sorted plots of CCALD-OPCs differentiated from CCALD-iPSCs after co-staining with anti-A2B5 and anti-NG2 antibodies, as indicated.

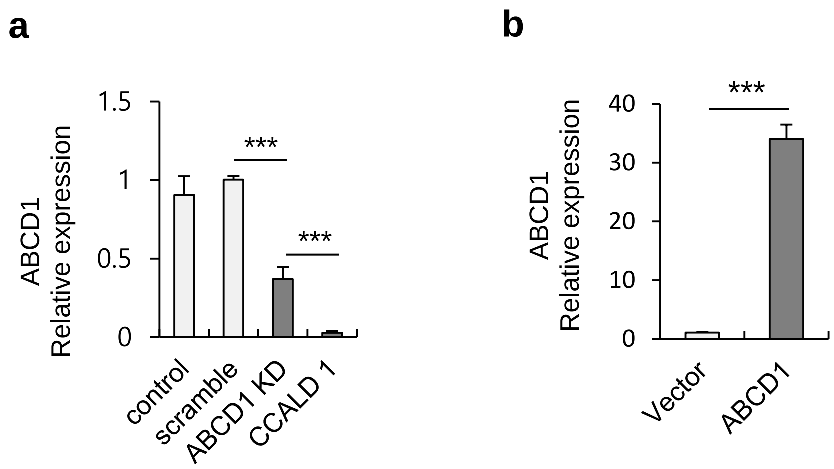

Supplementary Figure 4. **Quantification of ABCD1 mRNA production after knockdown or ectopic expression of ABCD1**. (**a**) Control fibroblasts were untransfected or transfected with scrambled or ABCD1-targeting siRNA. (**b**) CCALD-fibroblasts were infected with lentiviral particles expressing control vector or *ABCD1.* (**a,b**) Relative expression of *ABCD1* mRNA was then determined. a, ***P<0.001 (n = 6); b, ***P<0.001 (n = 5).

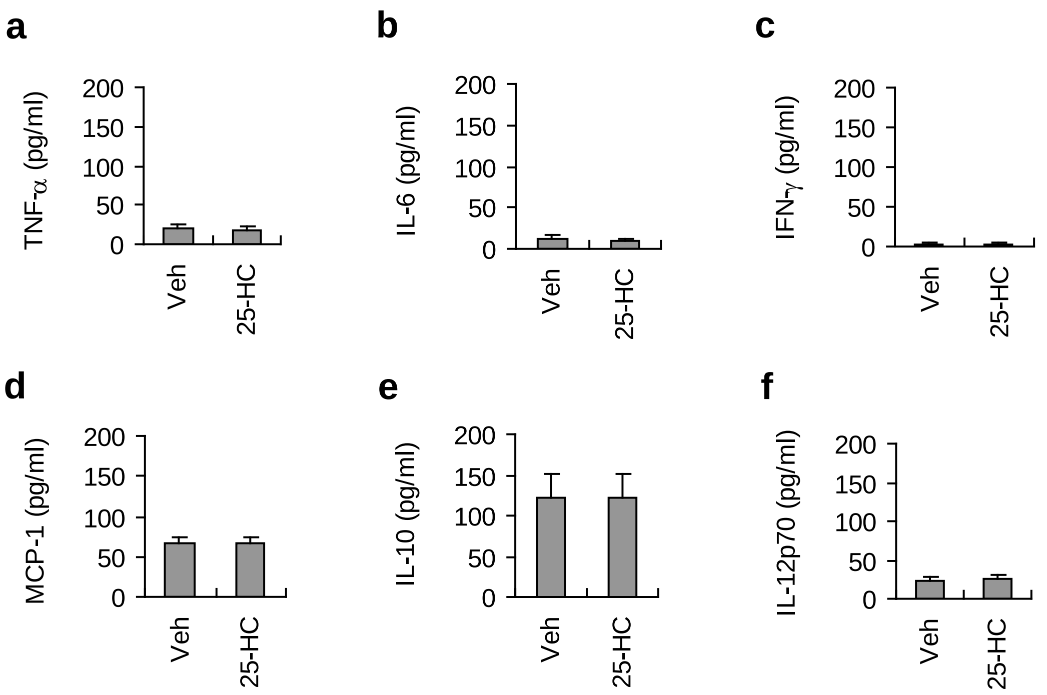

Supplementary Figure 5. Quantification of inflammatory cytokines in brain homogenates after stereotaxic injection of 25-HC. (**a-f**) Vehicle or 25-HC (100 μM) were stereotaxically injected into the corpus callosum of mouse brains. Three days after injection, mice were sacrificed, and the brain homogenates were assayed for inflammatory cytokines using a mouse inflammation cytometric bead array kit. (*n* = 8)

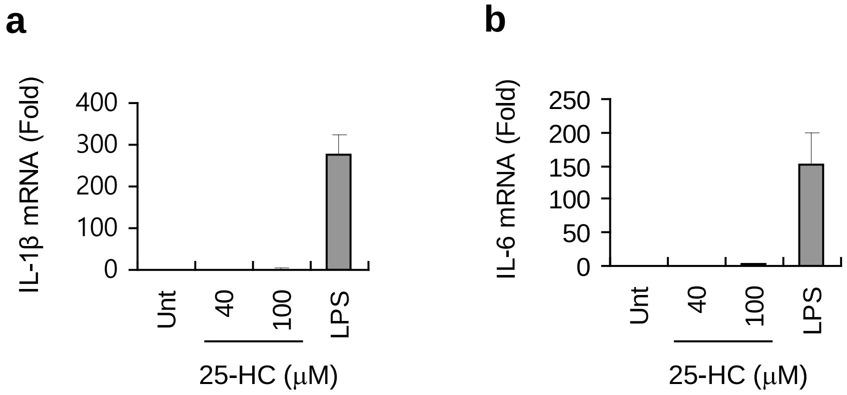

Supplementary Figure 6. 25-HC does not induce the mRNA production of IL-1β and IL-6. (**a,b**) Mouse BMDMs were treated with 25-HC at indicated concentrations for 10 h or LPS (0.25 μg ml^{-1}) for 4 h. mRNA expression of IL-1β and IL-6 was quantified by quantitative real-time PCR. (*n* = 3)

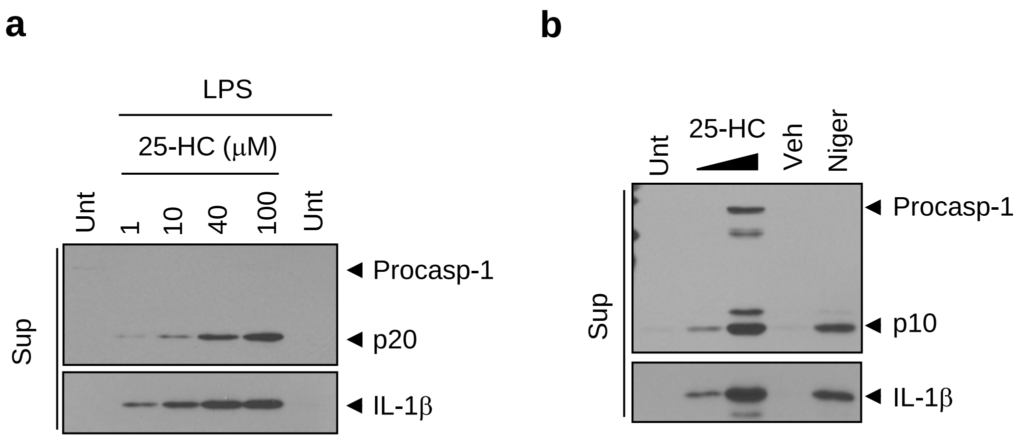

Supplementary Figure 7. 25-HC promotes robust caspase-1 activation. (**a**) Immunoblots of culture supernatants from the primary mixed glial cultures treated with 25-HC at indicated concentrations for 10 h in the presence of LPS (last 4 h). (**b**) Immunoblots of culture supernatants from PMA-differentiated THP-1 cells treated with 25-HC (50 or 100 μM, 10 h) or treated with nigericin (5 μM, 1 h).

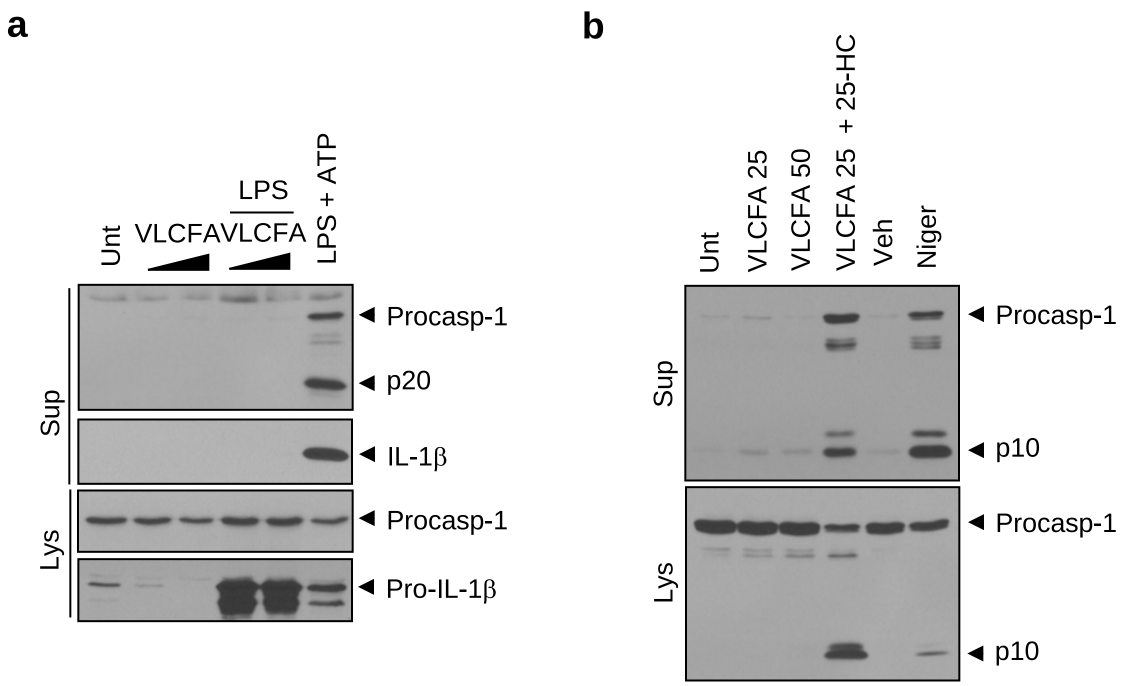

Supplementary Figure 8. VLCFA does not promote the caspase-1 activation. (**a**) Immunoblots of supernatant fractions (Sup) or cellular lysates (Lys) of mouse primary mixed glial cells treated with VLCFA (10 or 25 μM, 8 h) in the presence of LPS, or treated with LPS followed by ATP. (**b**) Immunoblots of PMA-differentiated THP-1 cells treated with VLCFA (25 or 50 μM, 10 h) in the presence of 25-HC (100 μM), or with nigericin (5 μM, 1 h).

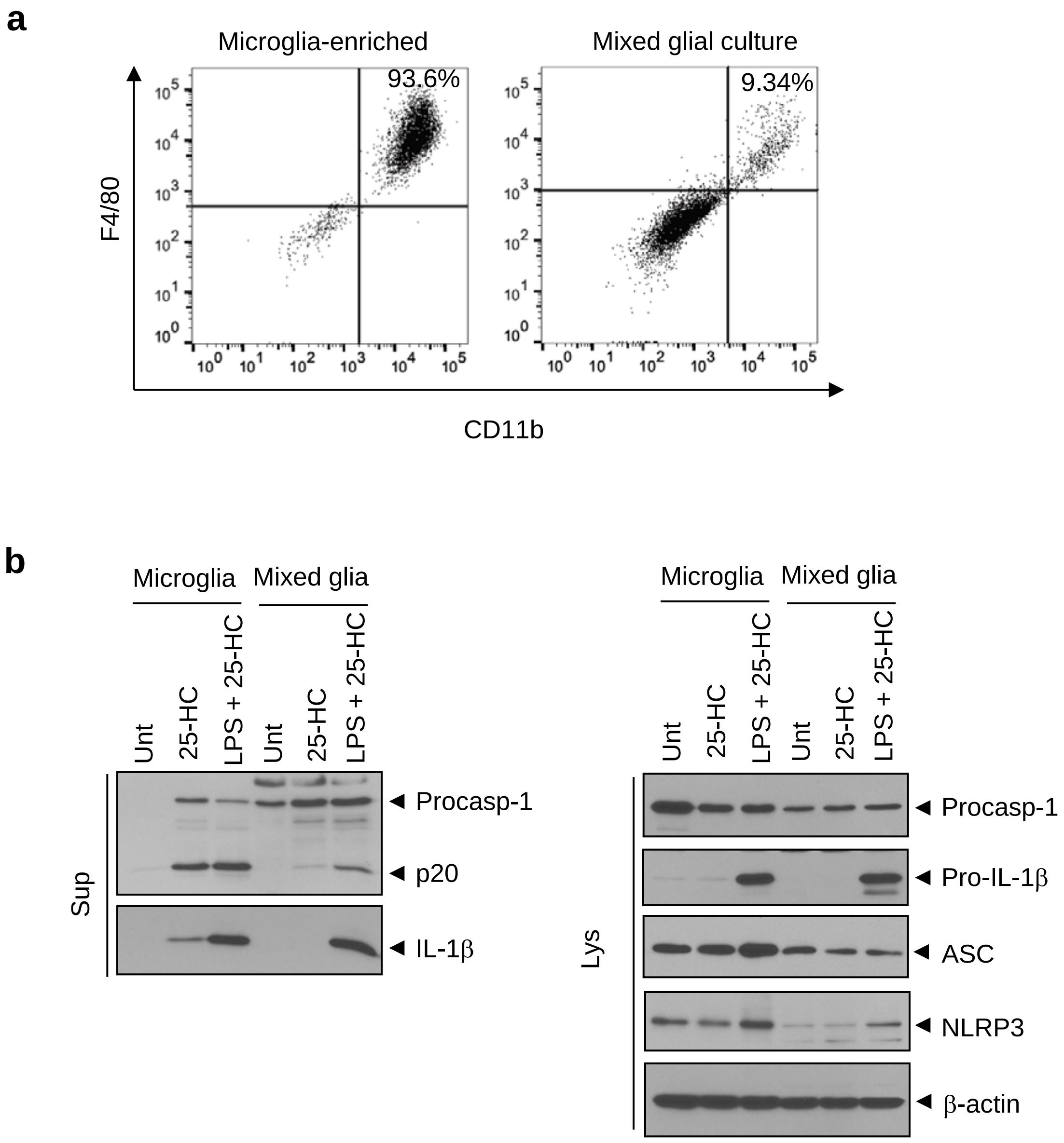

Supplementary Figure 9. 25-HC promotes inflammasome activation in mouse primary microglia and mixed glial cells. (**a**) Flow cytometric analysis of microglia-enriched population or mixed glial cultures after co-staining with anti-CD11b-PE and anti-F4/80-APC antibodies. (**b**) Immunoblots of culture supernatants (Sup) or cellular lysates (Lys) from microglia or mixed glial cells untreated or treated with 25-HC (100 μM, 10 h) in the presence or absence of LPS (4 h).

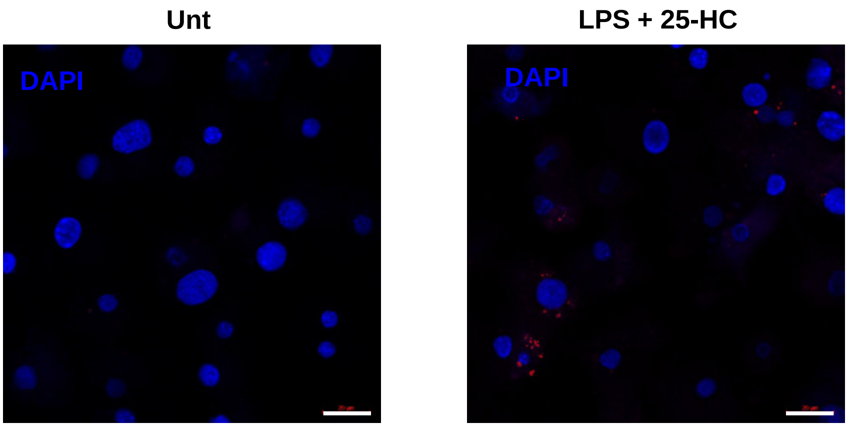

Supplementary Figure 10. 25-HC triggers a molecular association between NLRP3 and ASC by Proximity ligation assay. Mouse BMDMs were untreated or treated with 25-HC (80 μM, 10 h) in the presence of LPS (final 4 h). Proximity ligation assays were then performed. The red color indicates the proximity of NLRP3 and ASC. DAPI represents nuclei (blue). Scale bars, 20 μm.

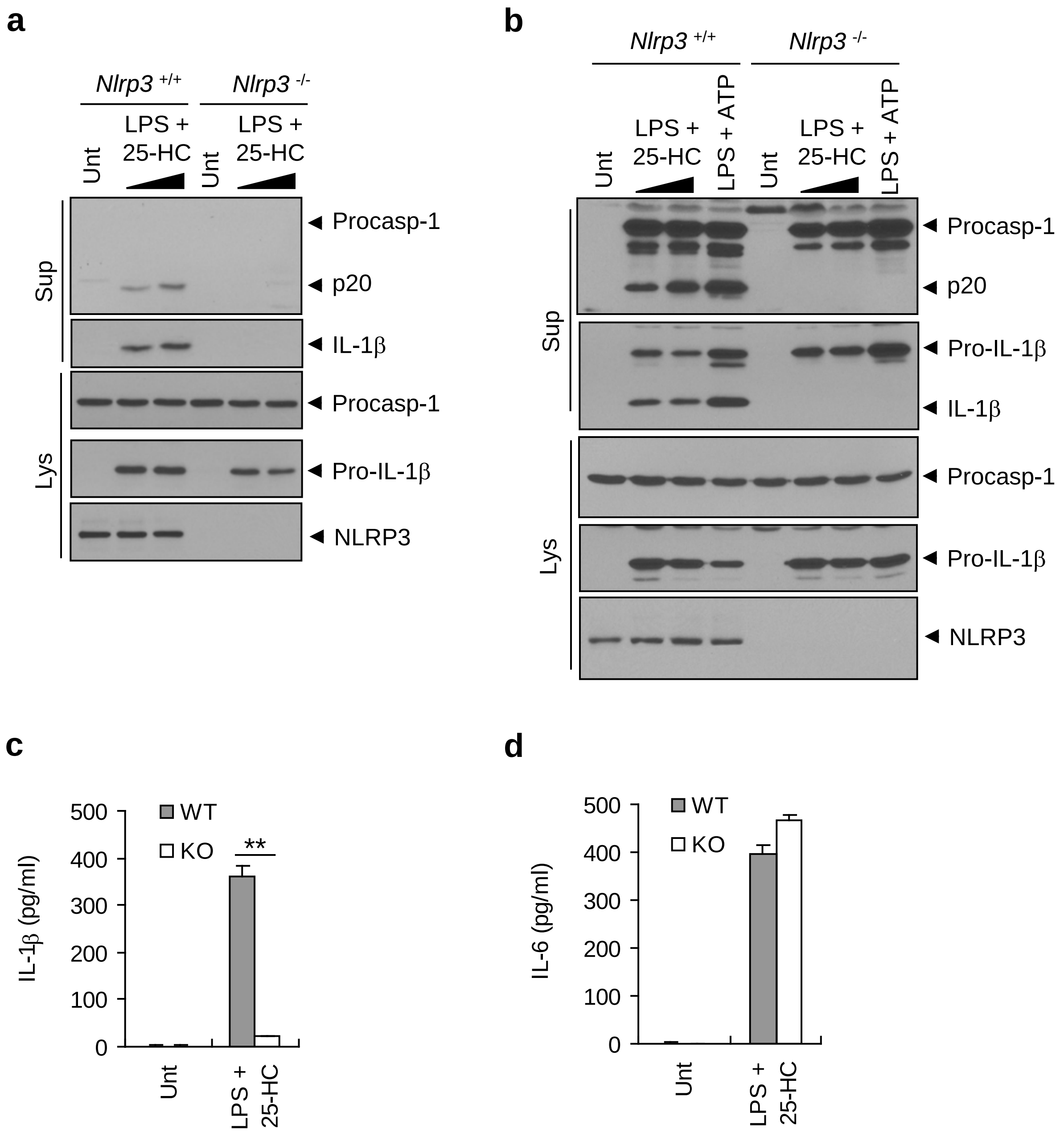

Supplementary Figure 11. 25-HC promotes NLRP3-dependent caspase-1/IL-1β signaling. (**a**) *Nlrp3*$^{+/+}$ or *Nlrp3*$^{-/-}$ microglial cells were treated with 25-HC (20 or 50 μM, 10 h) in the presence of LPS and culture supernatants (Sup) or cellular lysates (Lys) were analyzed by immunoblot. (**b**) Immunoblots of supernatant fractions (Sup) or cellular lysates (Lys) from *Nlrp3*$^{+/+}$ or *Nlrp3*$^{-/-}$ mixed glial cells treated with 25-HC (40 or 100 μM, 10 h) in the presence of LPS (last 4 h), or treated with LPS, followed by ATP. (**c,d**) Quantification of IL-1β (c) or IL-6 (d) from supernatant fractions of *Nlrp3*$^{+/+}$ (WT) or *Nlrp3*$^{-/-}$ (KO) BMDMs treated with 25-HC (50 μM, 10 h) in the presence of LPS (last 4 h). Unt, untreated. ***P*<0.01 (*n* = 3).

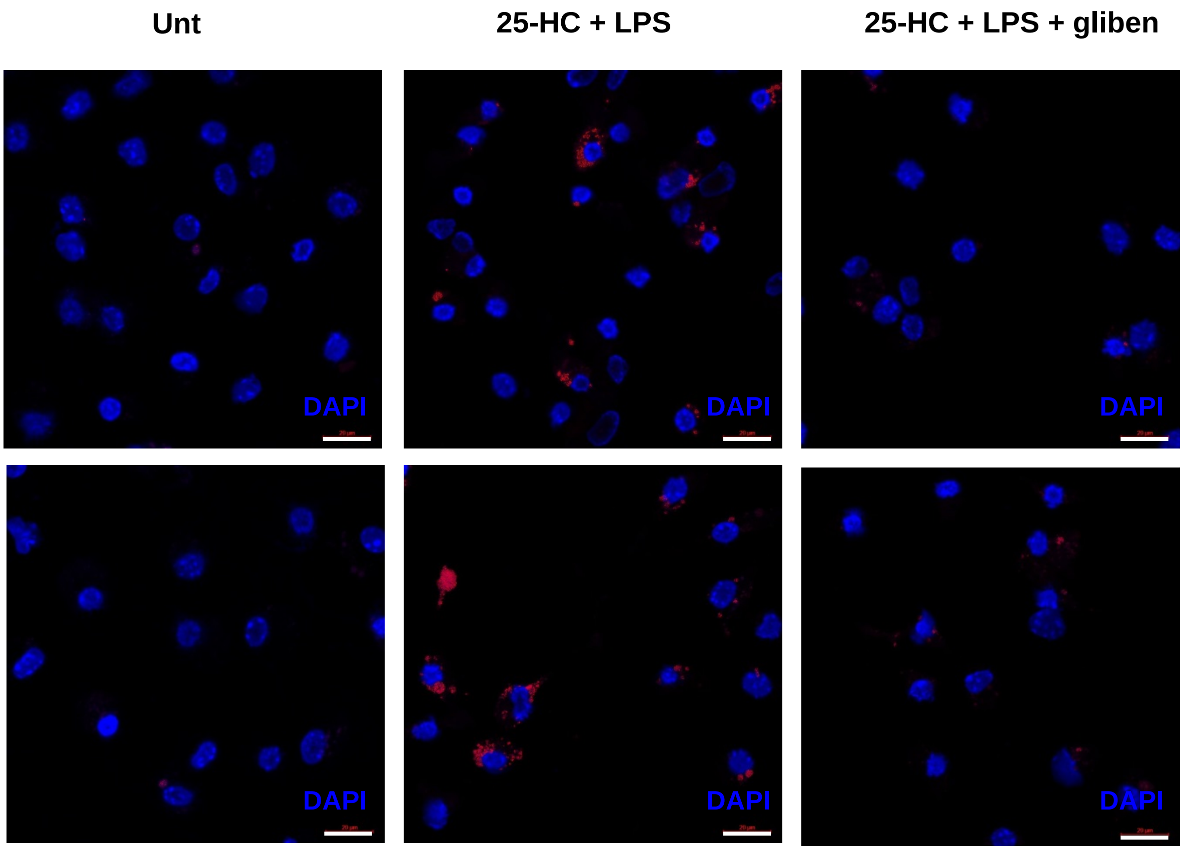

Supplementary Figure 12. Inhibition of potassium efflux attenuates the molecular assembly of NLRP3 with ASC. Mouse microglial cells were untreated or treated with 25-HC (80 μM, 10 h) together with glibenclamide (50 μM, 10 h) in the presence of LPS (last 4 h). Proximity ligation assays were then performed. The red color indicates the proximity of NLRP3 and ASC. Representative images of two independent samples are shown. DAPI represents nuclei (blue). Scale bars, 20 μm.

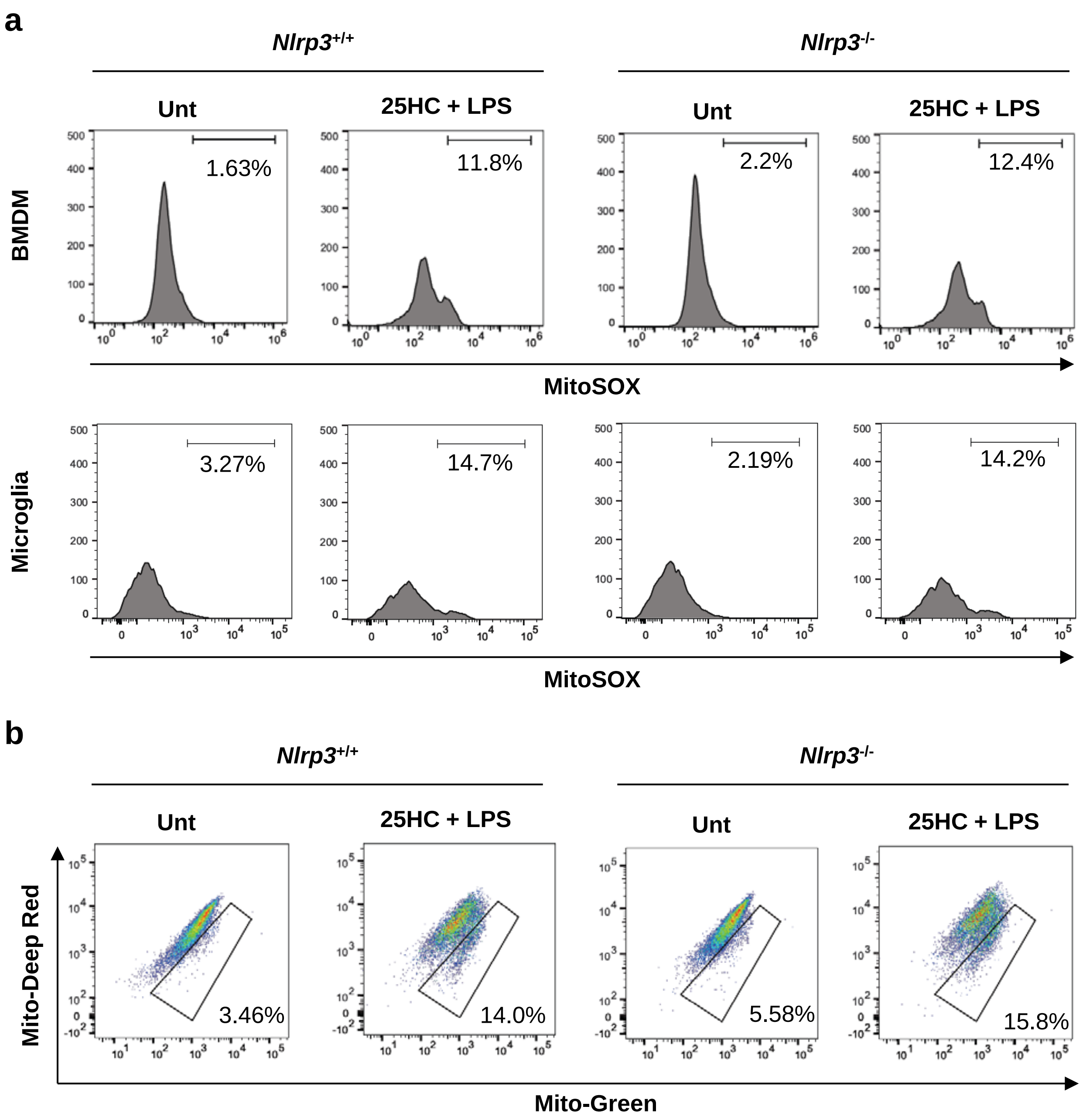

Supplementary Figure 13. 25-HC induces mitochondrial ROS production and mitochondrial damage in an NLRP3-independent manner. (**a,b**) *Nlrp3*$^{+/+}$ or *Nlrp3*$^{-/-}$ mouse BMDMs (a, upper panel; b) or microglia (a, lower panel) were treated with 25-HC (50 μM, 8 h) in the presence of LPS (last 3 h). Cells were stained with MitoSOX (a) or MitoTracker Deep Red and MitoTracker Green (b), and analyzed by flow cytometer. The populations of MitoSOX-positive cells (a) and of damaged mitochondria-containing cells (b) are indicated.

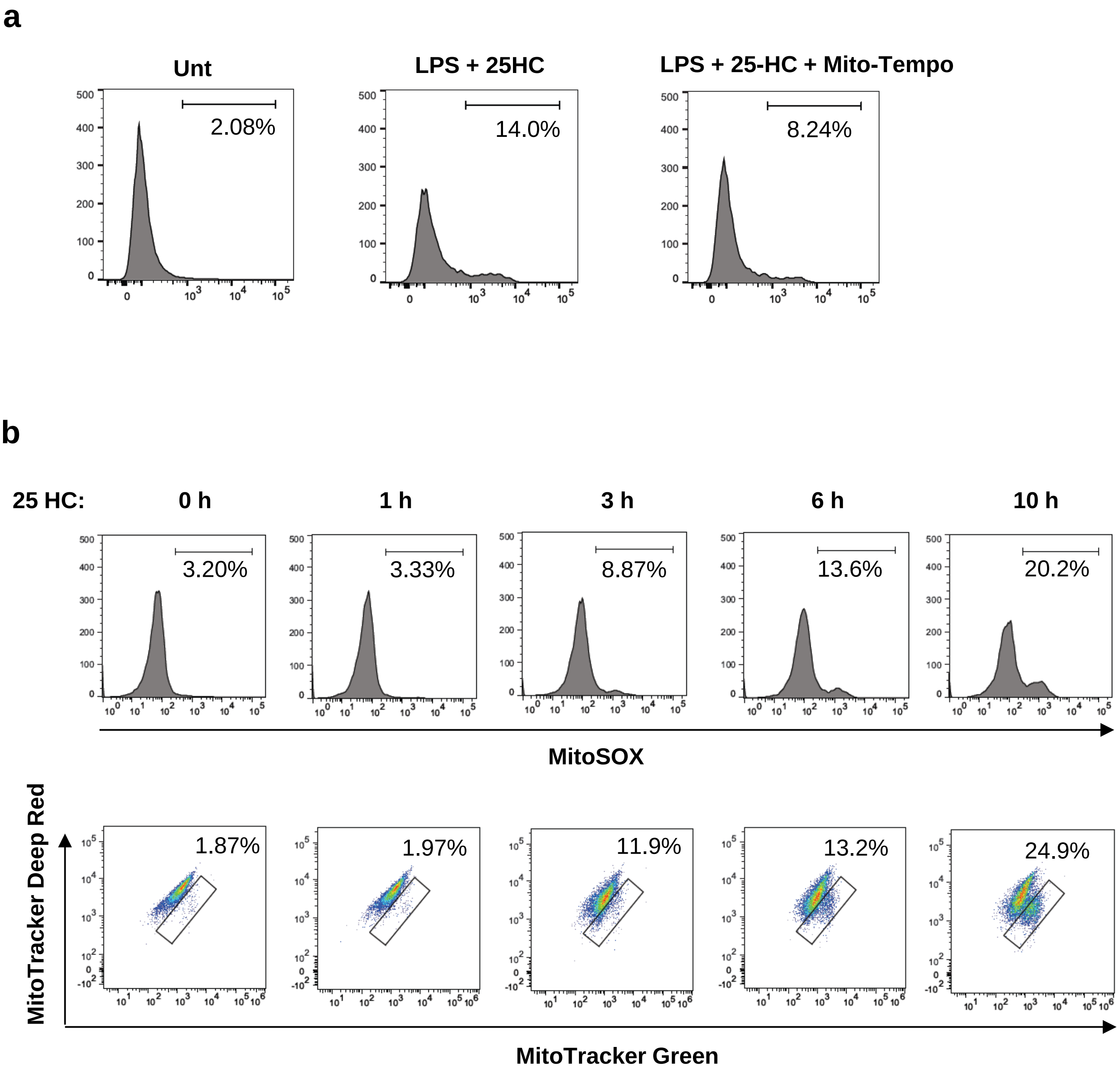

Supplementary Figure 14. 25-HC induces time-dependent mitochondrial ROS production and mitochondrial damage. (**a**) Mouse BMDMs were primed with LPS (0.25 μg ml^{-1}, 3 h), followed by treatment with 25-HC (50 μM) in the presence of Mito-Tempo (200 μM) for 6 h. Cells were stained with MitoSOX, then analyzed by flow cytometry. (**b**) Mouse BMDMs were treated with 25-HC (50 μM) for the indicated times (1~10 h) in the presence of LPS (0.25 μg ml^{-1}, final 1 or 3 h). Cells were then stained with MitoSOX (upper panel) or costained with MitoTracker Deep Red and MitoTracker Green (lower pannel), and analyzed by flow cytometry. The populations of MitoSOX-positive cells (upper) and of damaged mitochondria-containg cells (lower) are indicated.

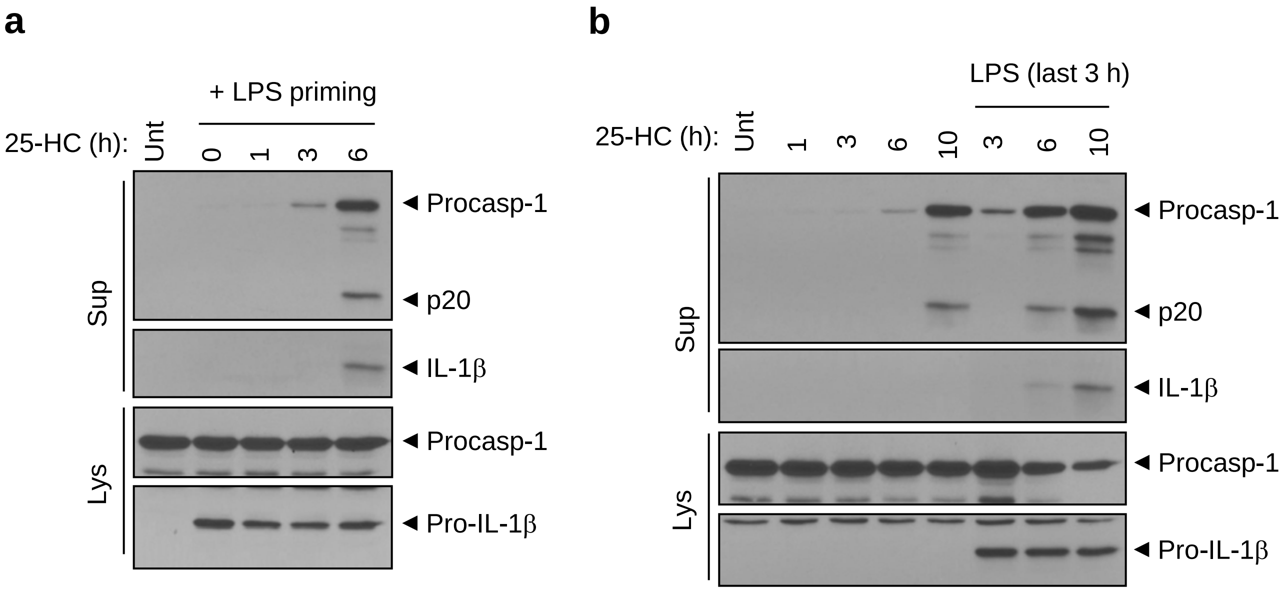

Supplementary Figure 15. Time-dependent activation of caspase-1 by 25-HC. (**a**) Mouse BMDMs were primed with LPS (0.25 μg ml^{-1}, 3 h), followed by treatment with 25-HC (50 μM) for the indicated times (1~6 h). (**b**) Mouse BMDMs were treated with 25-HC (50 μM) for the indicated times in the presence or absence of LPS (0.25 μg ml^{-1}, final 3 h). (**a,b**) Culture supernatants (Sup) or cellular lysates (Lys) were immunoblotted with the indicated antibodies.

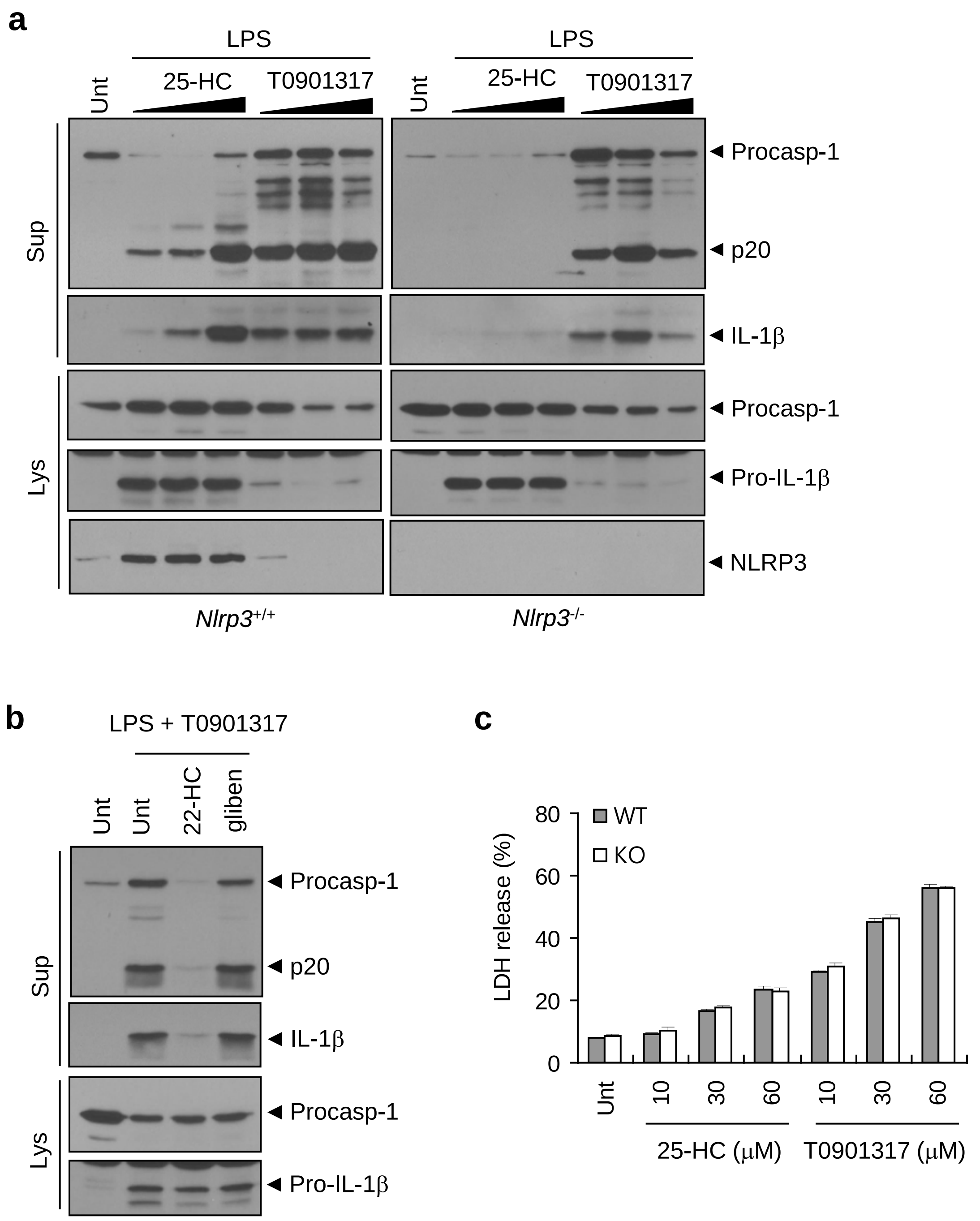

Supplementary Figure 16. TO90317, a LXR ligand, induces the activation of caspase-1 and IL-1β in an NLRP3-independent manner. (**a**) *Nlrp3*$^{+/+}$ or *Nlrp3*$^{-/-}$ mouse BMDMs were primed with LPS (0.25 μg ml^{-1}, 3 h), followed by treatment with 25-HC or TO901317 (20~60 μM) for 6 h. (**b**) Mouse BMDMs were primed with LPS (3 h), followed by TO901317 (80 μM) in the presence of 22-HC (100 μM) or glibenclamide (50 μM) for 6 h. (**a, b**) Culture supernatants (Sup) or cellular lysates (Lys) were immunoblotted with the indicated antibodies. (**c**) *Nlrp3*$^{+/+}$ (WT) or *Nlrp3*$^{-/-}$ (KO) mouse BMDMs were primed with LPS (0.25 μg ml^{-1}, 3 h), followed with the treatment of 25-HC or TO901317 (10~60 μM) for 6 h). Cell death was then determined by extracellular release of lactate dehydrogenase (LDH). (*n* = 3).

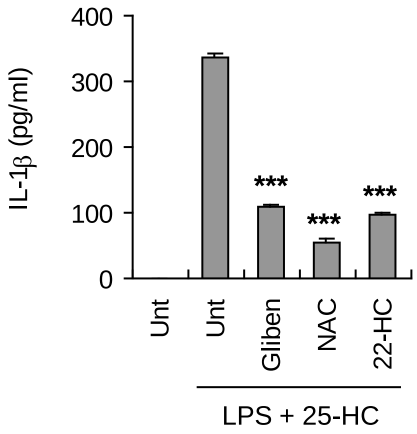

Supplementary Figure 17. Inhibition of potassium efflux, mitochondrial ROS production, and LXR signaling attenuates the 25-HC-triggered secretion of IL-1β. Mouse BMDMs were primed with LPS (0.25 μg ml^{-1}, 3 h), followed by treatment with 25-HC (80 μM) in the presence of glibenclamide (50 μM), NAC (20 mM), or 22-HC (100 μM) for 6 h. The level of IL-1β in the culture supernatants was quantified by ELISA. Asterisks indicate the significant differences compared with the samples treated with LPS + 25-HC (2nd lane). ***P<0.001 (n = 3)

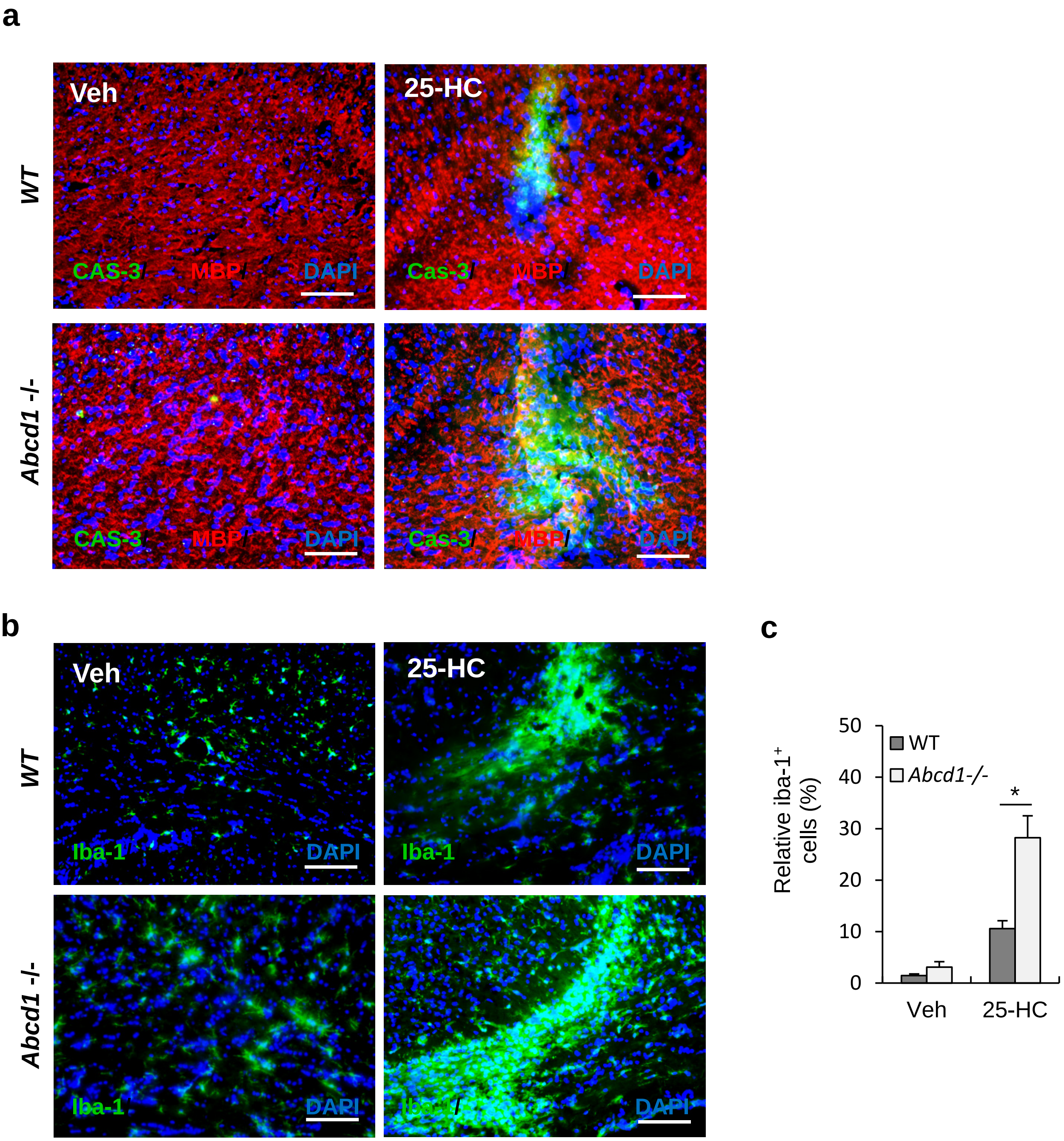

Supplementary Figure 18. Deficiency of *Abcd1* confers increased susceptibility to stereotaxic injection of 25-HC into mouse brains. (**a**) Representative images of brain sections immunostained with anti-active caspase-3 (green), anti-myelin basic protein (MBP, red) antibodies, and DAPI (blue) after bilateral stereotaxic injection of vehicle or 25-HC (100 μM) into the brains of *Abcd1*$^{+/+}$ (WT) or *Abcd1*$^{-/-}$ mice. Scale bars, 100 μm. (**b**) Immunohistochemical staining of coronal brain sections with anti-Iba-1 antibody (green) and DAPI (blue) after bilateral stereotaxic injection of 25-HC (100 μM) or vehicle into the brains of of *Abcd1*$^{+/+}$ (WT) or *Abcd1*$^{-/-}$ mice. Scale bars, 100 μm. (**c**) Quantification of Iba-1-positive microglia (green) in the confocal images of vehicle- or 25-HC-injected brains of *Abcd1*$^{+/+}$ (WT) or *Abcd1*$^{-/-}$ mice. (n = 6)

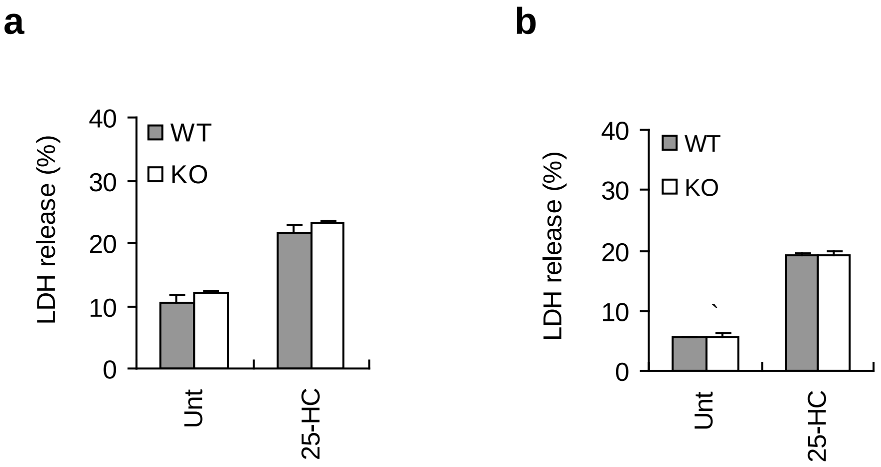

Supplementary Figure 19. 25-HC induces NLRP3-independent cell death. (**a, b**) *Nlrp3*$^{+/+}$ (WT) or *Nlrp3*$^{-/-}$ (KO) mouse BMDMs (a) or mixed glial cells (b) were treated with 25-HC (80 μM) for 6 h. Cell death was then determined by extracellular release of lactate dehydrogenase (LDH). (*n* = 3)

Supplementary Figure 20. Original full images of immunoblots.

Figure 4a

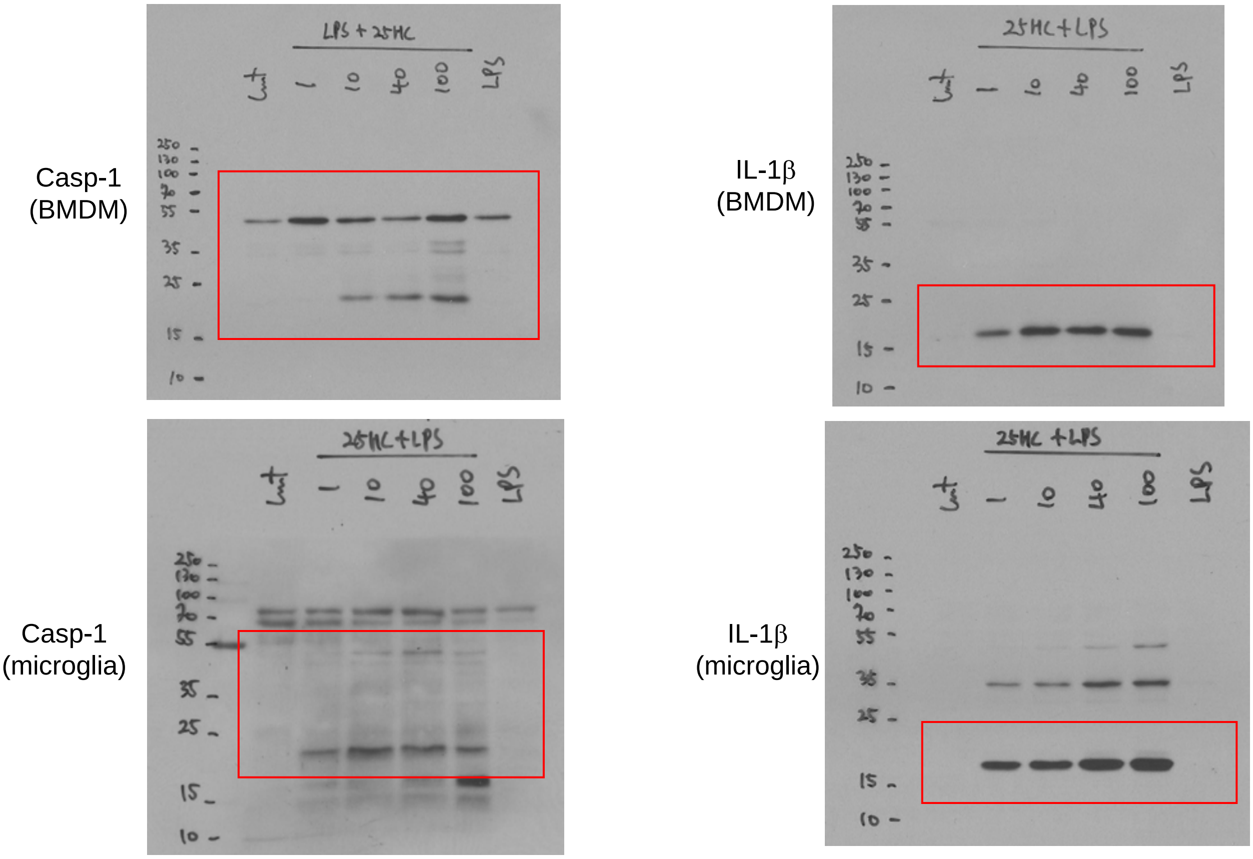

Figure 4b

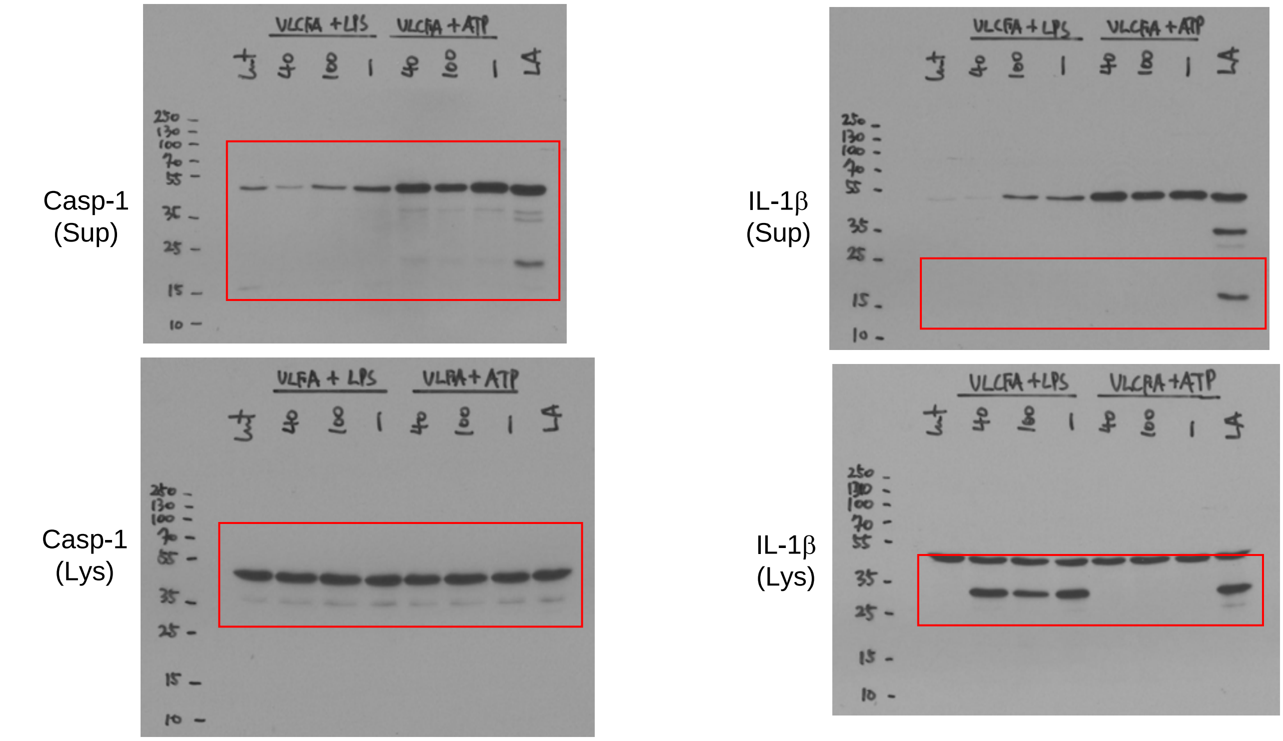

Figure 4c

ASC
(Pel + DSS)

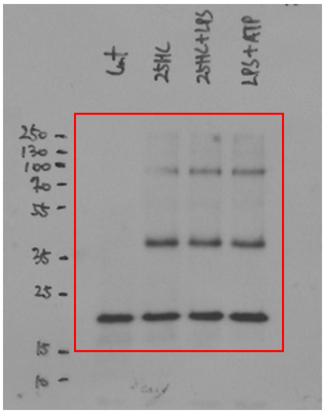

ASC
(Lys)

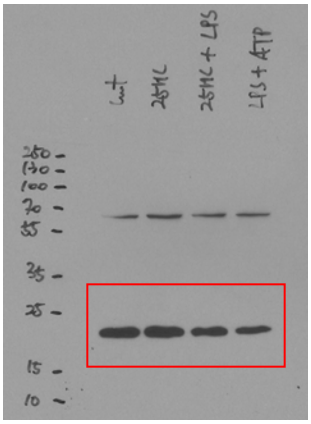

Figure 4f

Casp-1
(Sup)

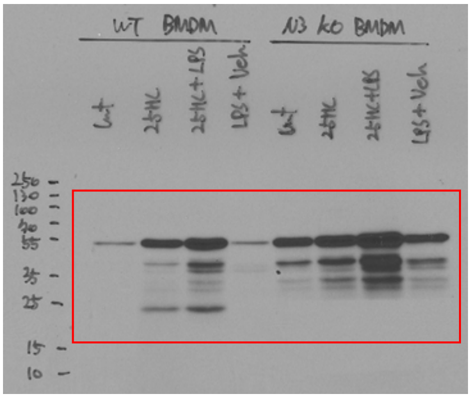

IL-1β
(Sup)

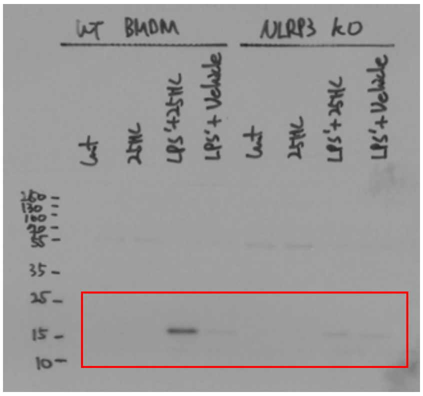

Casp-1
(Lys)

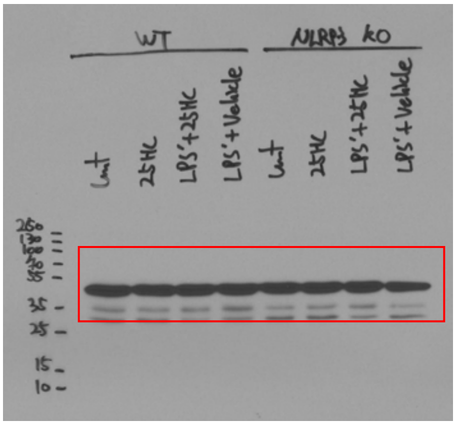

IL-1β
(Lys)

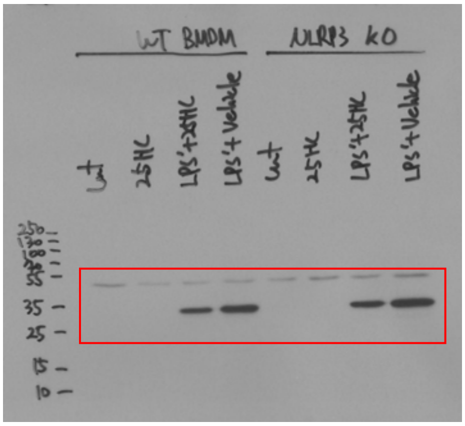

NLRP3
(Lys)

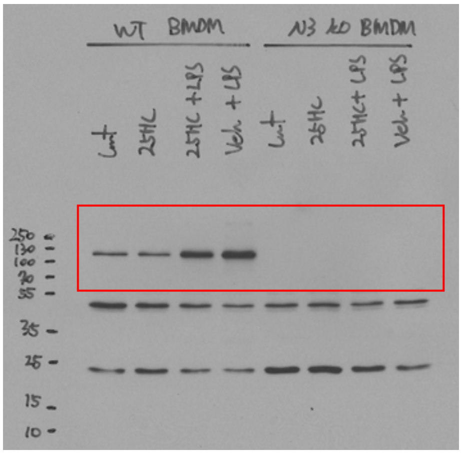

Figure 4g

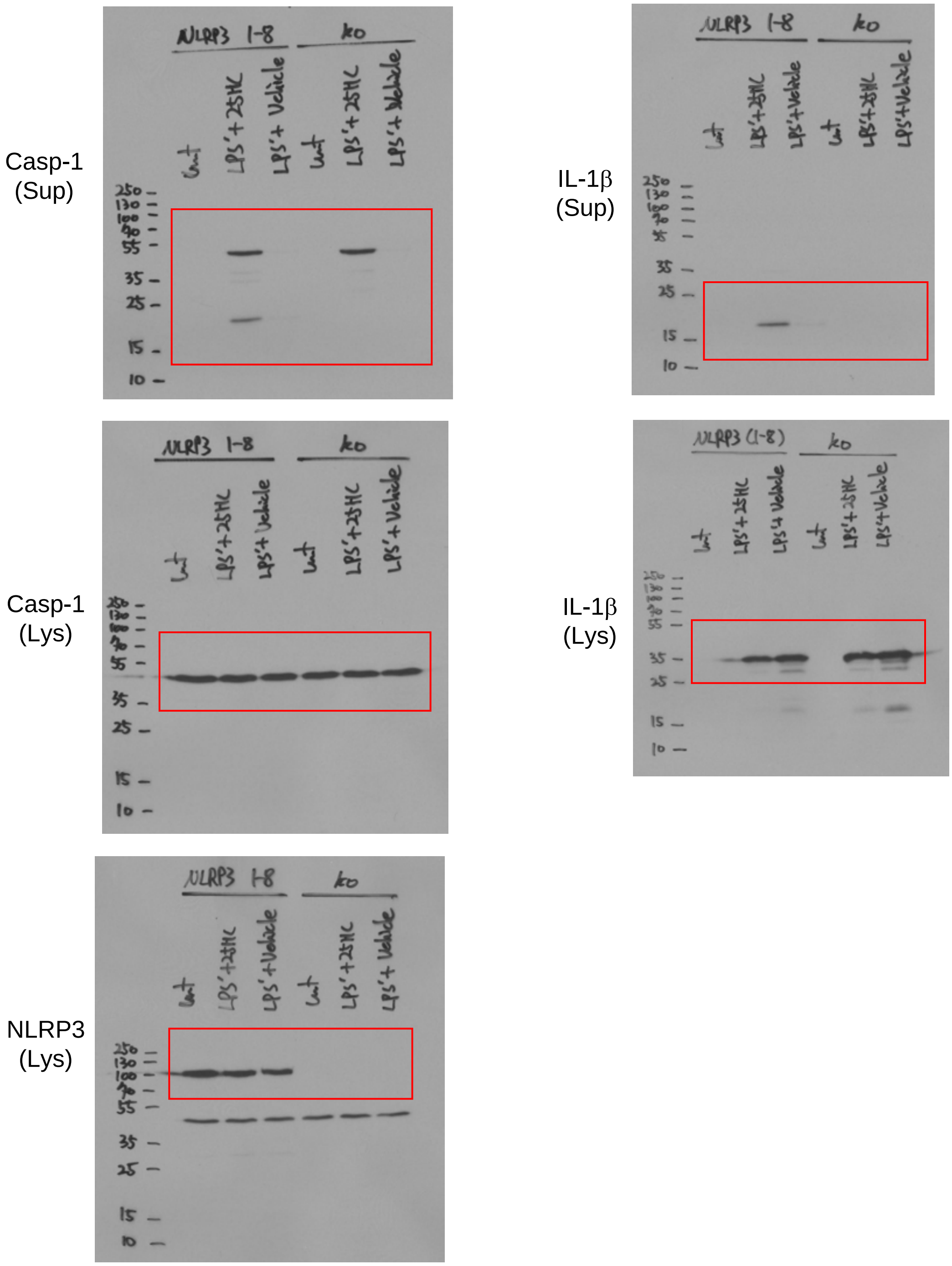
Casp-1
(Sup)
IL-1β
(Sup)
Casp-1
(Lys)
IL-1β
(Lys)
NLRP3
(Lys)
NLRP3 1-8
ko
Unt
LPS'+25HC
LPS'+Vehicle
250
130
100
70
55
35
25
15
10

Figure 5a

Casp-1
(Sup)

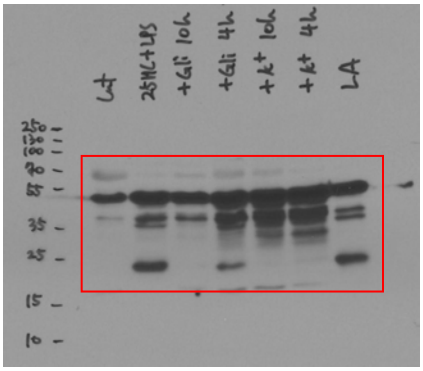

IL-1β
(Sup)

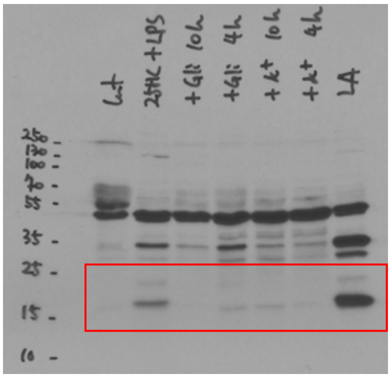

Casp-1
(Lys)

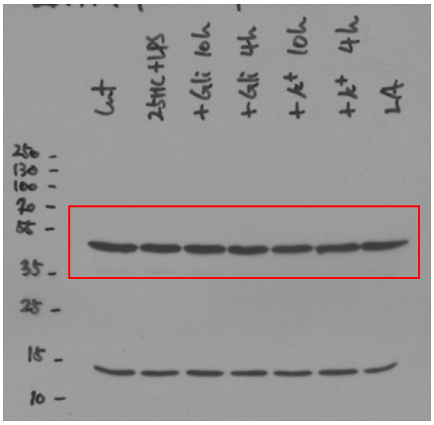

IL-1β
(Lys)

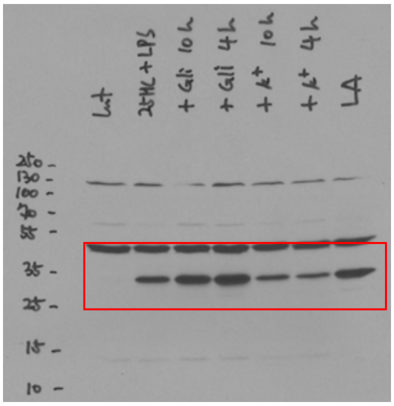

Figure 5b

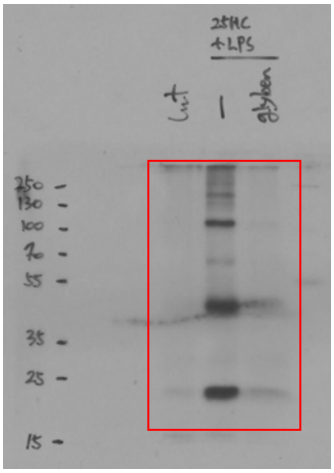

ASC
(Pel + DSS)

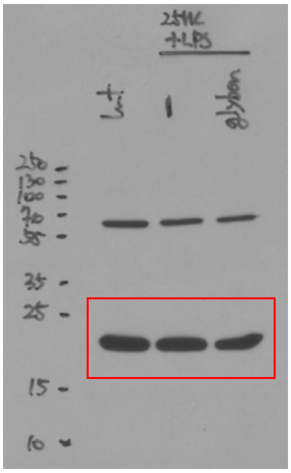

ASC
(Lys)

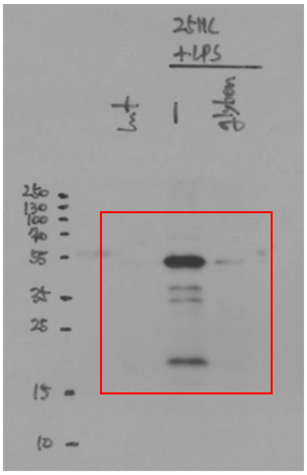

Casp-1
(Sup)

Figure 5e

Casp-1 (Sup)

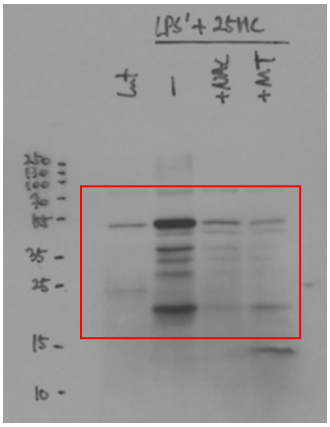

IL-1β (Sup)

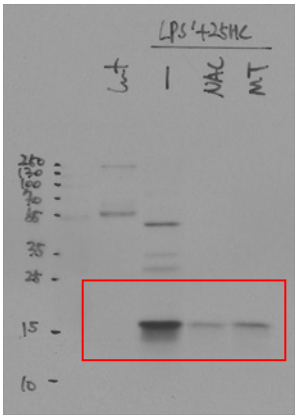

Casp-1 (Lys)

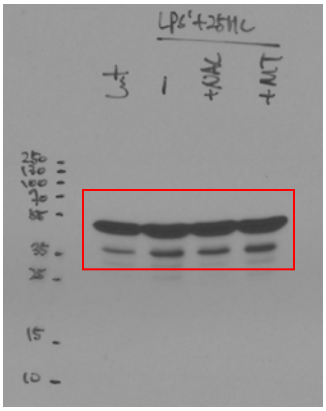

IL-1β (Lys)

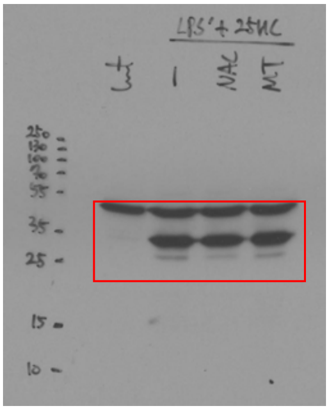

Figure 5f

ASC (Pel + DSS)

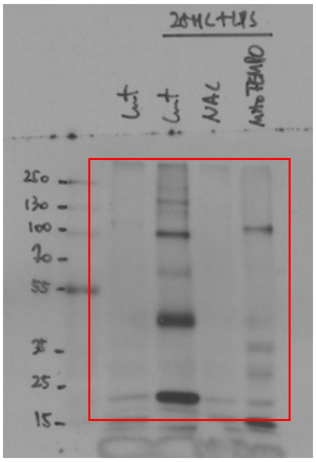

ASC (Lys)

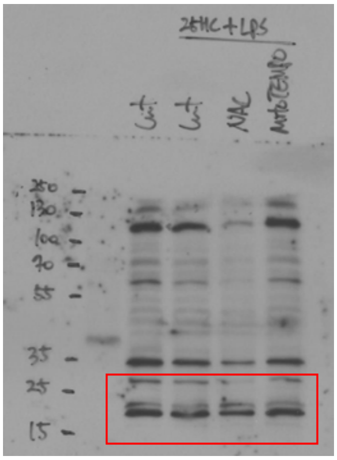

Figure 5g

Casp-1
(Sup)

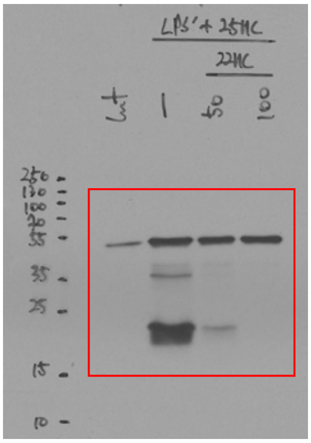

IL-1β
(Sup)

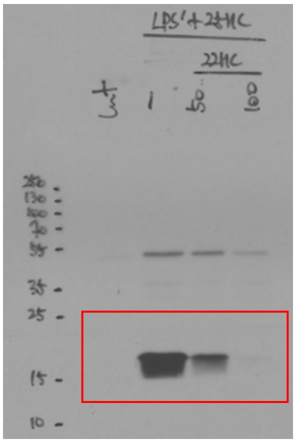

Casp-1
(Lys)

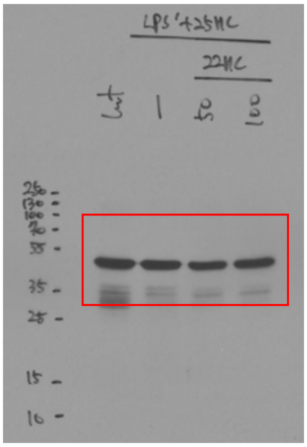

IL-1β
(Lys)

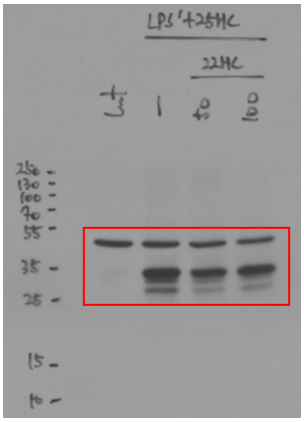

Figure 5h

Casp-1
(Sup)

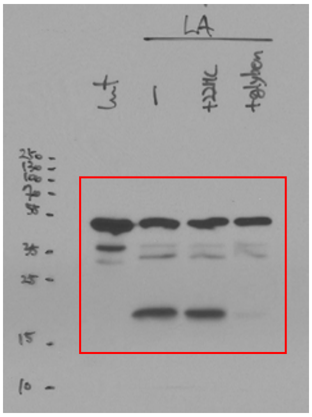

IL-1β
(Sup)

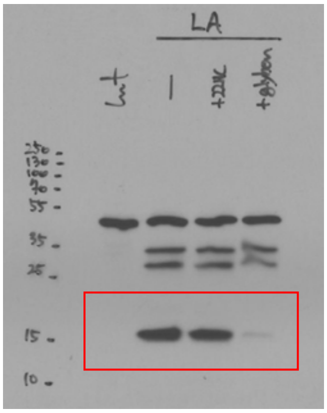

Casp-1
(Lys)

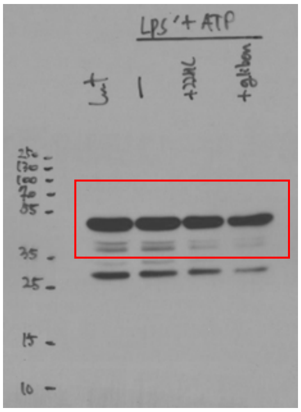

IL-1β
(Lys)

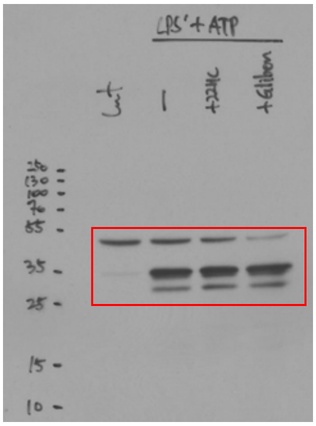

Figure 5i

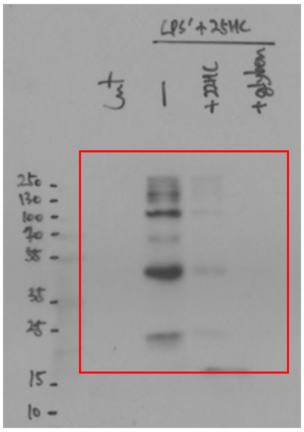

ASC
(Pel + DSS)

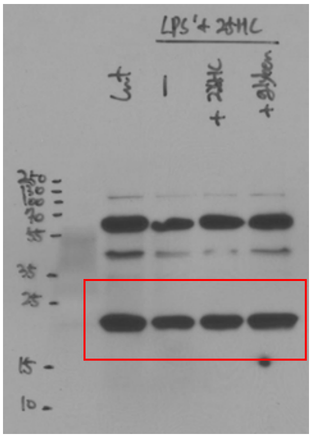

ASC
(Lys)

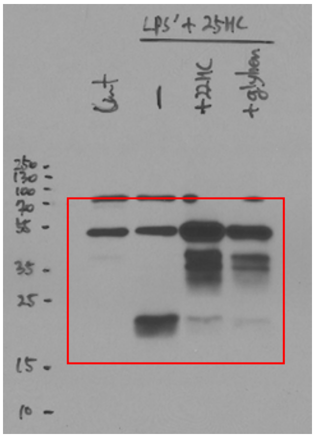

Casp-1
(Sup)

Supplementary Figure 7a

Casp-1
(Sup)

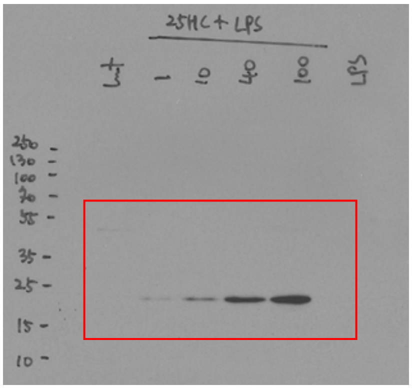

IL-1β
(Sup)

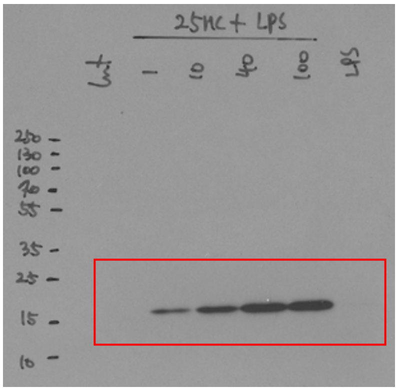

Supplementary Figure 7b

Casp-1
(Sup)

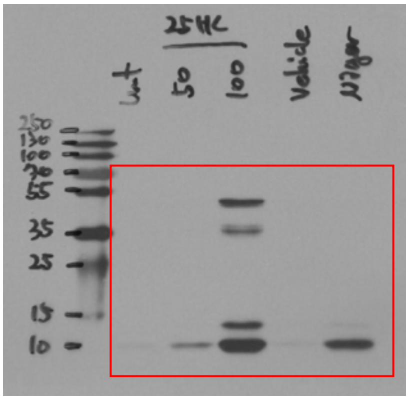

IL-1β
(Sup)

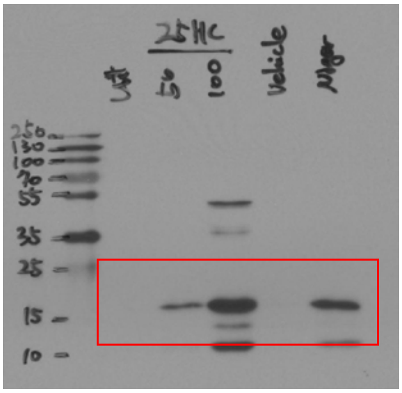

Supplementary Figure 8a

Casp-1
(Sup)

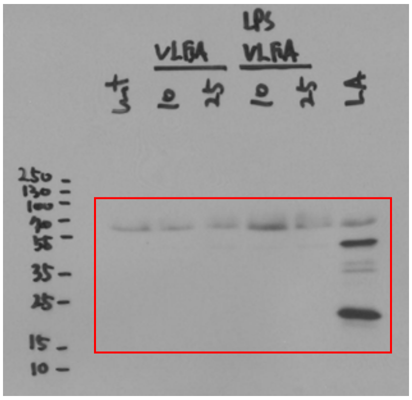

IL-1β
(Sup)

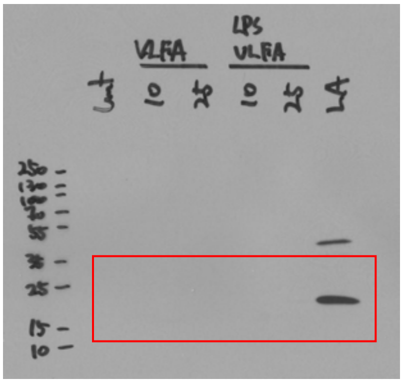

Casp-1
(Lys)

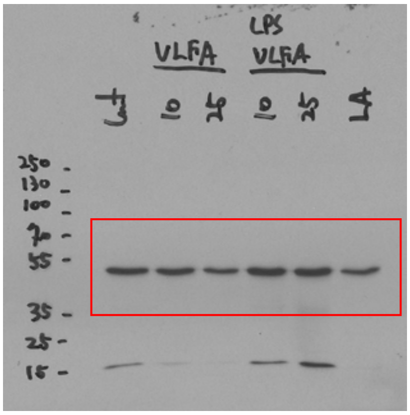

IL-1β
(Lys)

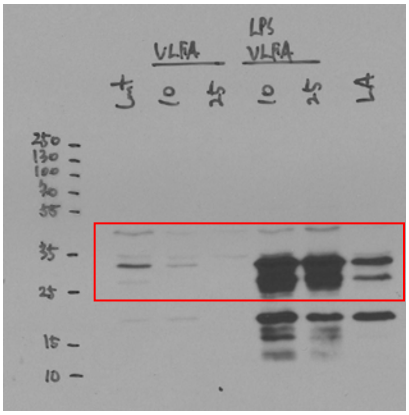

Supplementary Figure 8b

Casp-1
(Sup)

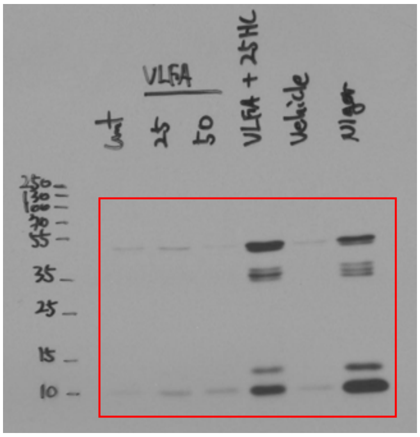

Casp-1
(Lys)

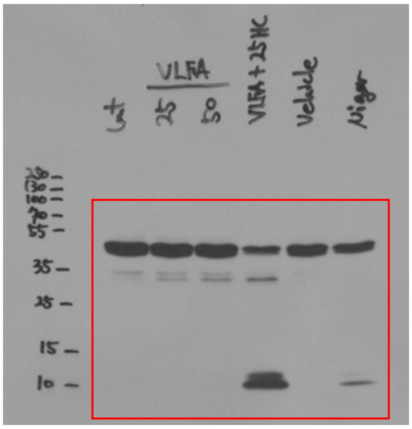

Supplementary Figure 9b

Casp-1
(Sup)

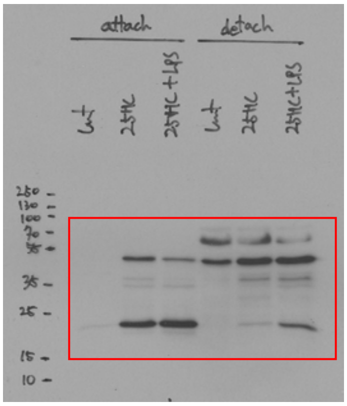

IL-1β
(Sup)

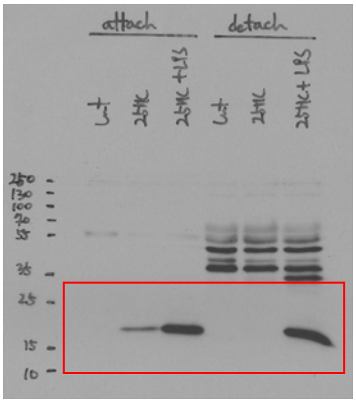

Casp-1
(Lys)

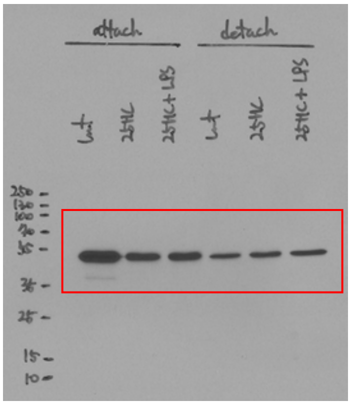

IL-1β
(Lys)

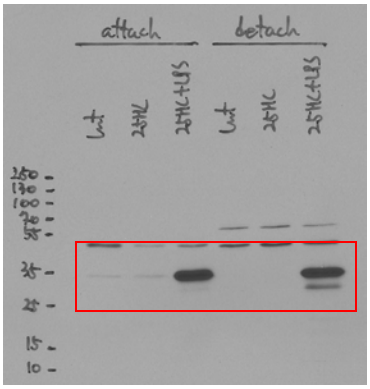

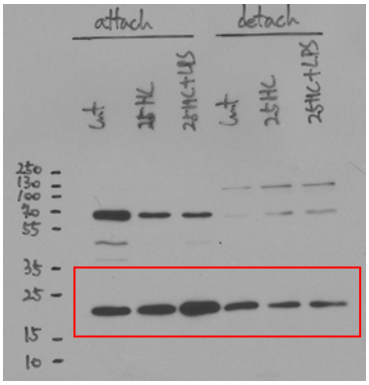

ASC
(Lys)

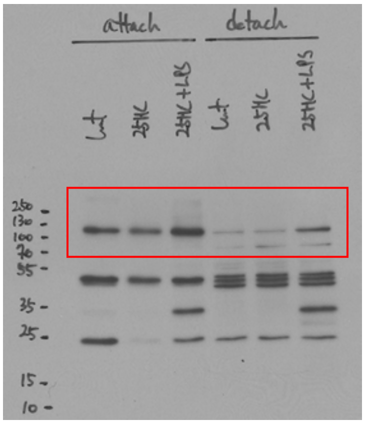

NLRP3
(Lys)

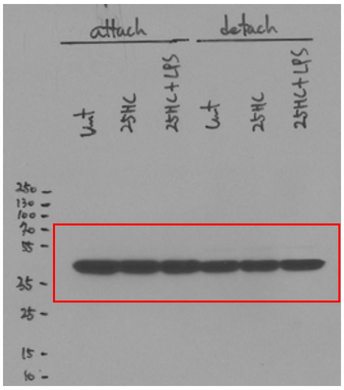

β-actin
(Lys)

Supplementary Figure 11a

Casp-1
(Sup)

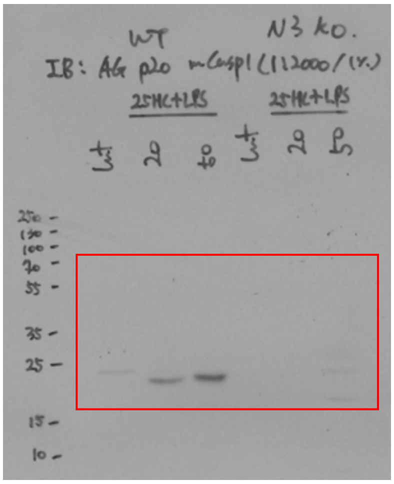

IL-1β
(Sup)

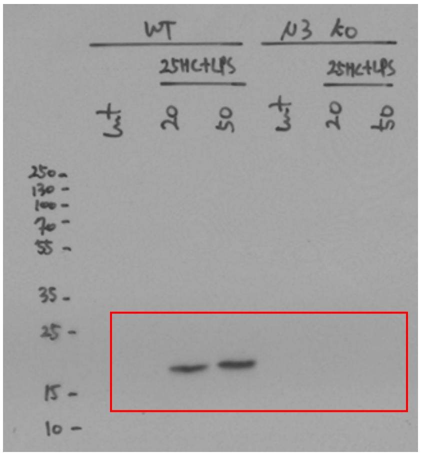

Casp-1
(Lys)

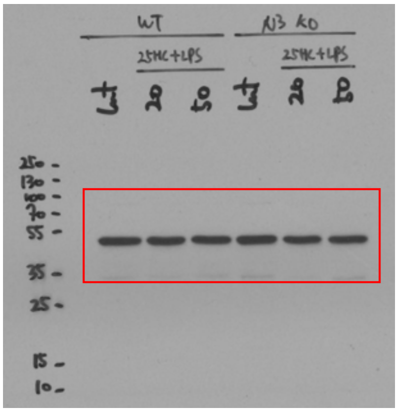

IL-1β
(Lys)

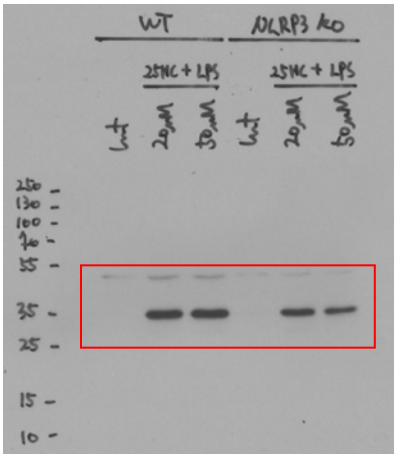

NLRP3
(Lys)

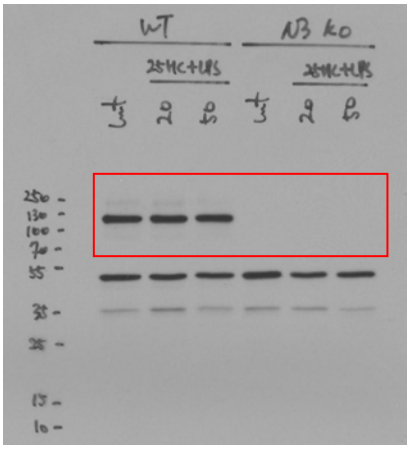

Supplementary Figure 11b

Casp-1
(Sup)

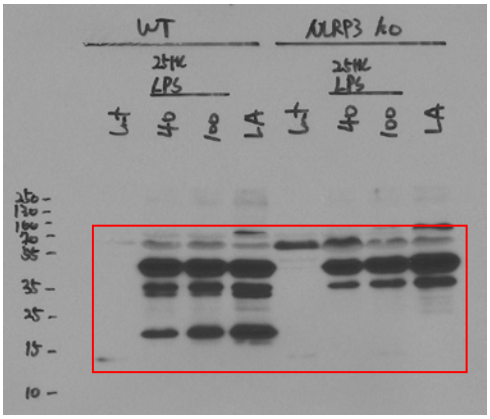

IL-1β
(Sup)

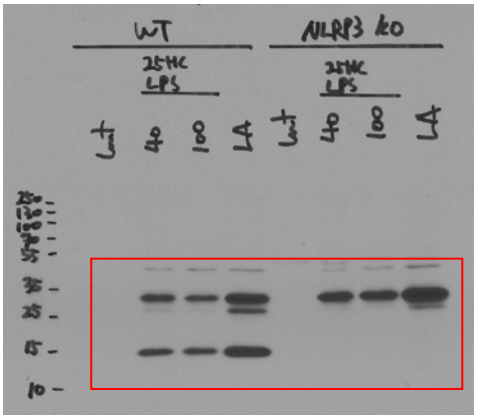

Casp-1
(Lys)

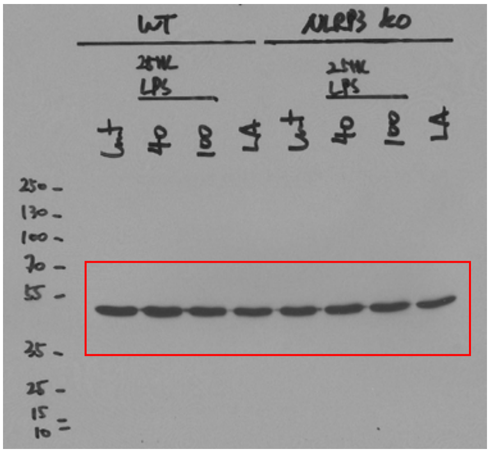

IL-1β
(Lys)

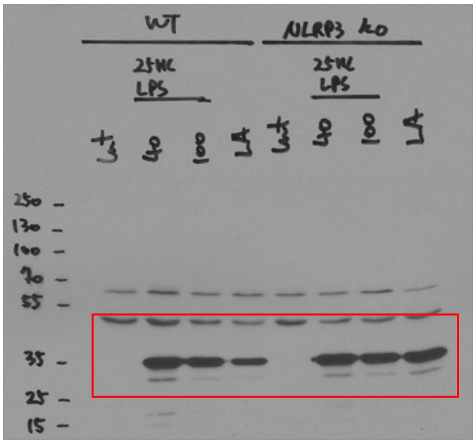

NLRP3
(Lys)

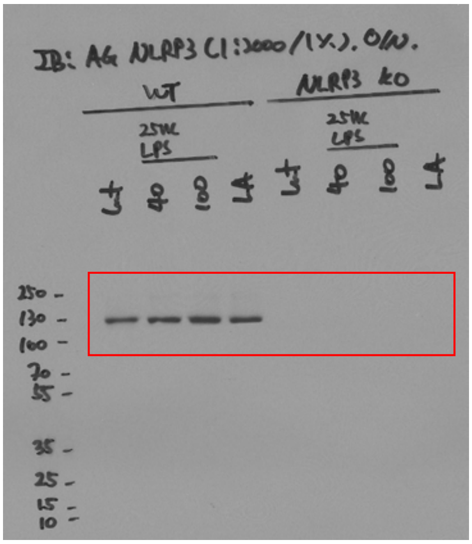

Supplementary Figure 15a

Casp-1
(Sup)

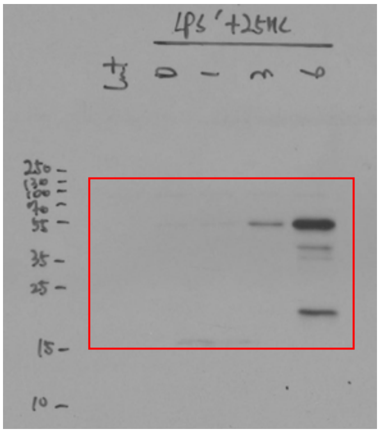

IL-1β
(Sup)

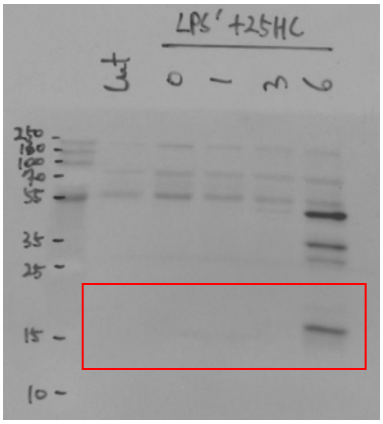

Casp-1
(Lys)

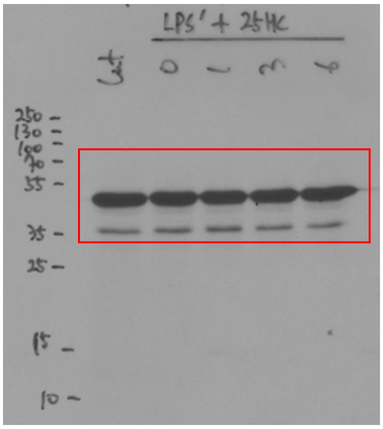

IL-1β
(Lys)

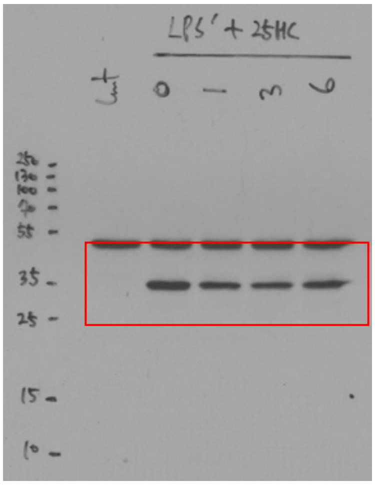

Supplementary Figure 15b

Casp-1
(Sup)

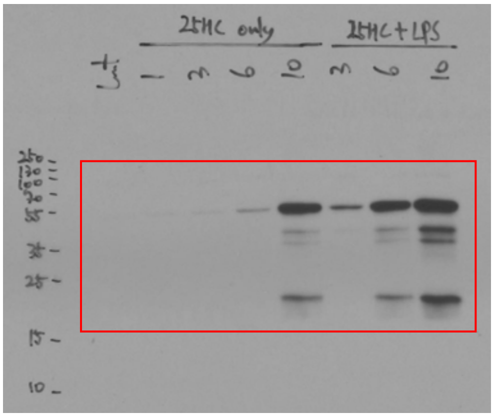

IL-1β
(Sup)

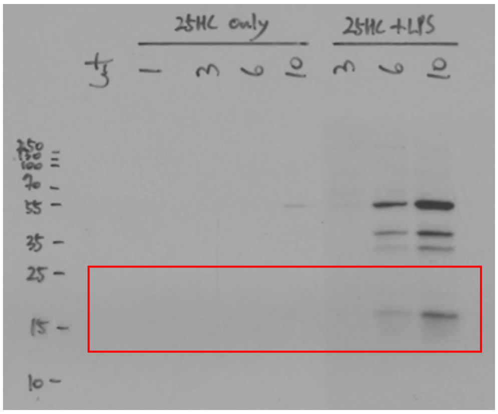

Casp-1
(Lys)

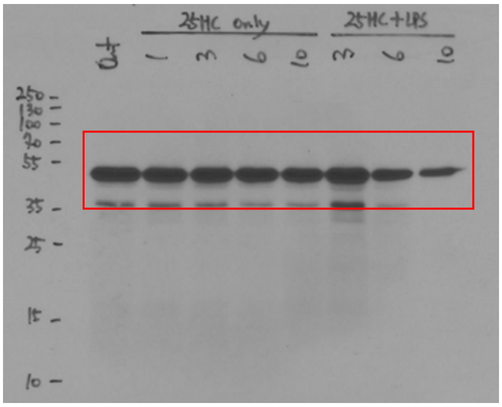

IL-1β
(Lys)

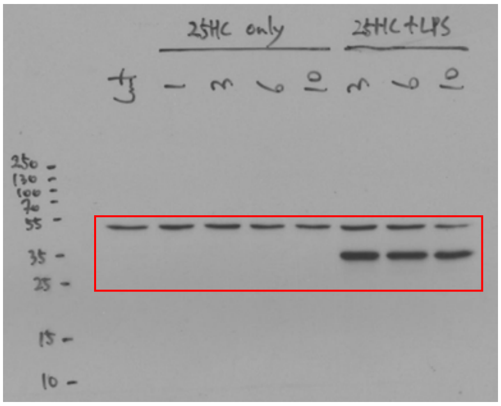

<u>Supplementary Figure 16a (*Nlrp3*$^{+/+}$)</u>

Casp-1
(Sup)

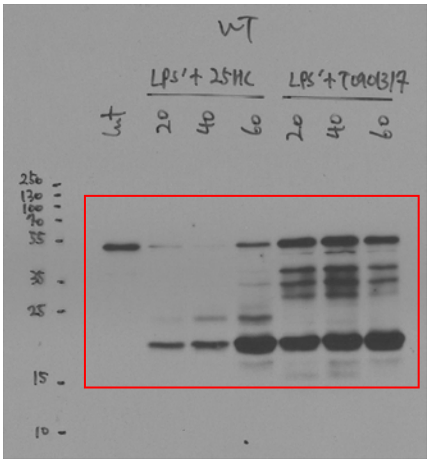

IL-1β
(Sup)

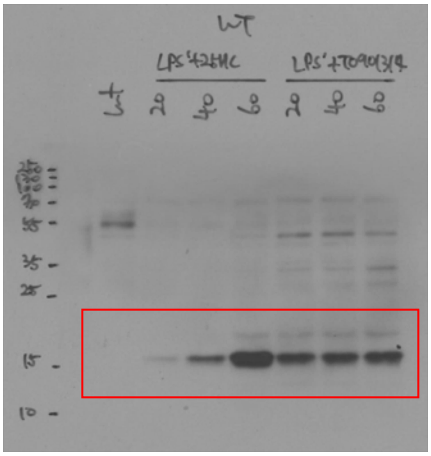

Casp-1
(Lys)

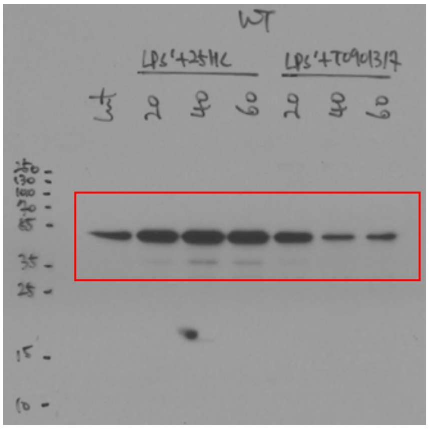

IL-1β
(Lys)

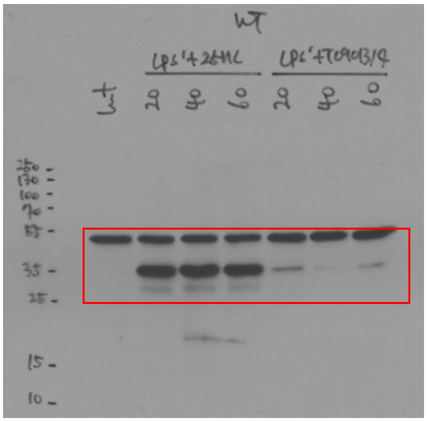

NLRP3
(Lys)

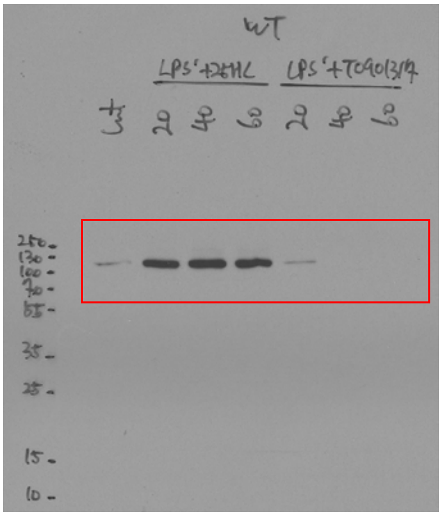

Supplementary Figure 16a (*Nlrp3*$^{-/-}$)

Casp-1
(Sup)

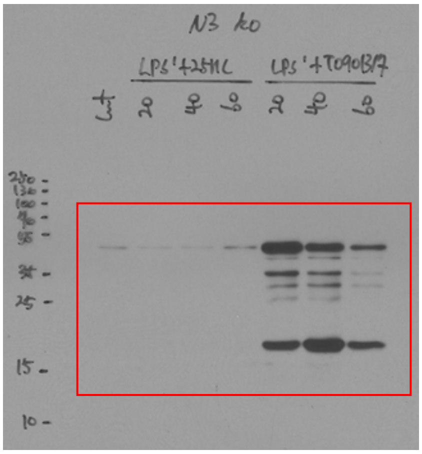

IL-1β
(Sup)

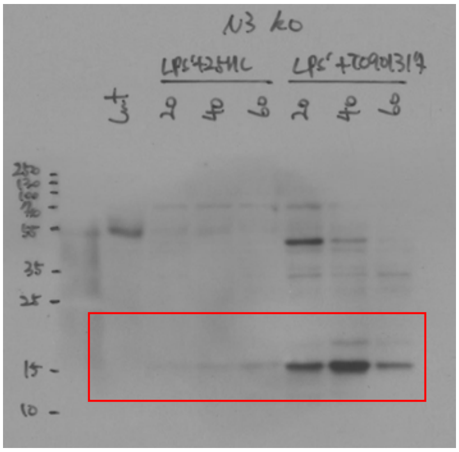

Casp-1
(Lys)

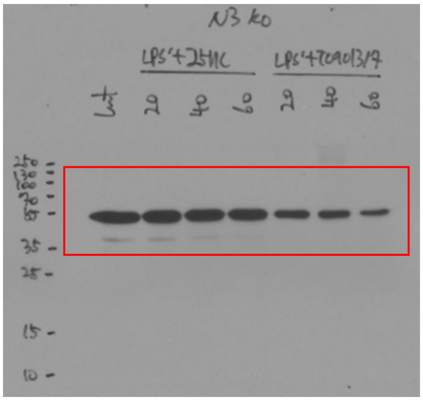

IL-1β
(Lys)

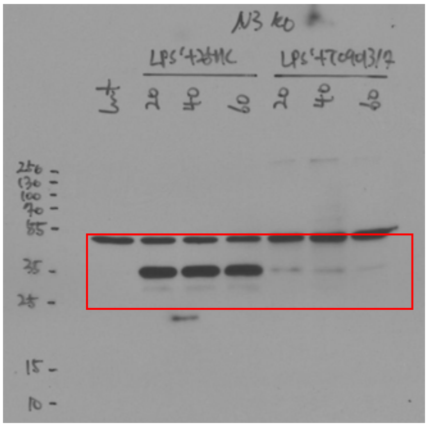

NLRP3
(Lys)

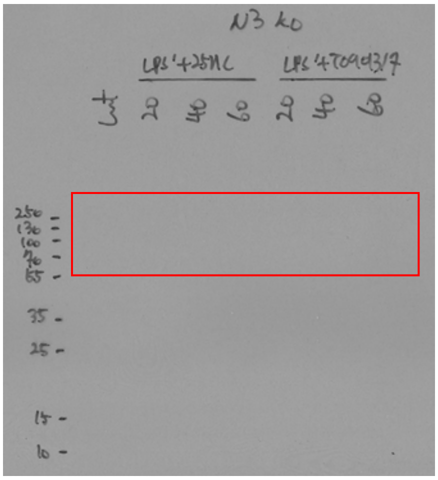

Supplementary Figure 16b

Casp-1
(Sup)

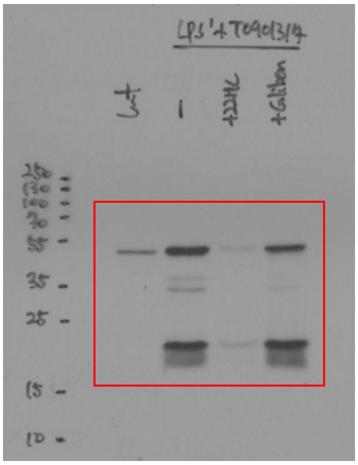

IL-1β
(Sup)

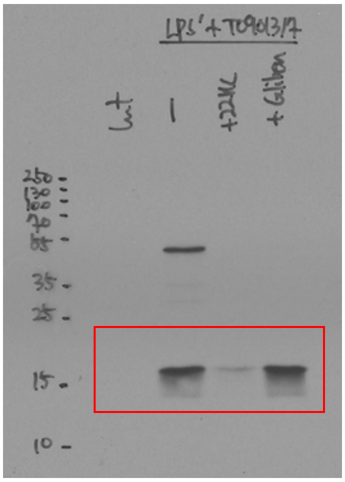

Casp-1
(Lys)

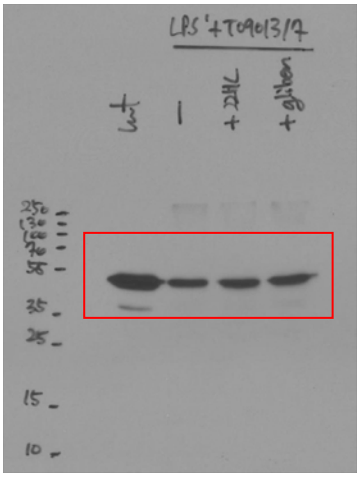

IL-1β
(Lys)

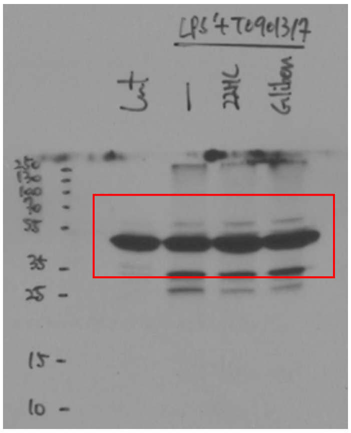

Supplementary Table 1. Microarray analysis of oxysterol-producing enzymes in the AMN- or CCALD-iPSCs.

	**AMN-iPSCs/Control-iPSCs**		**CCALD-iPSCs/Control-iPSCs**	
	Fold Change	*p*-value	Fold Change	*p*-value
CYP46A1	-1.010071	0.7399682	-1.281197	0.02891906
CYP27A1	1.120609	0.5171332	1.144407	0.2783103

Supplementary Table 2. Clinical information of X-ALD patients.

	Race	Age (YR)	Sex	Phenotypic data
CCALD 1 (GM04496)	Caucasian	6	M	High proportion of fatty acids with a chain length of 26 found in fibroblasts; autopsy pathological findings in both the adrenal and central nervous system were those of ALD
CCALD 2 (Korean Patient-1)	Korean	28	M	Elevated C26:C22 fatty acid ratio in plasma; impaired auditory discrimination and difficulties in vision at the age of 8; primary involvement of the occipital white matter; progressive manifestations of both the adrenal and central nervous system were those of CCALD; a point mutation in intervening sequence 4 (−2a > g) (c.1394-2A>G(IVS4(-2)A>G))
CCALD 3 (GM04934)	Caucasian	7	M	Elevated C26:C22 fatty acid ratio in fibroblasts and plasma
CCALD 4 (GM04932)	Polynesian	10	M	Maori tribe, New Zealand; many affected individuals in pedigree; elevated C26: C22 fatty acid ratio in fibroblasts; no clinical evidence of adrenal insufficiency; only microscopic evidence observed at autopsy
AMN 1 (Korean Patient-2)	Korean	24	M	Elevated C26:C22 fatty acid ratio in plasma; MRI of the brain was normal; slowly progressive peripheral axonopathy; was wheel chair bound by the age of 23; clinical course characteristic of adrenomyeloneuropathy
AMN 2 (GM07675)	Caucasian	22	M	Adrenomyeloneuropathy; neurologically normal until the age of 20; urinary tract dysfunction and mild spasticity in legs since the age of 22; abnormal EEG with posterior slowing; elevated levels of C26 fatty acids in plasma and fibroblasts; positive family history
AMN 3 (GM17819)	Caucasian	32	M	Clinically affected; skin biopsy taken from the forearm; presented with adrenal insufficiency at the age of 24; slow decline in motor function with spastic weakness in the lower limbs associated with loss of vibration sense; MRI of the brain at the age of 30 was normal; began using a cane at the age of 28 and was wheel chair bound by the age of 30; clinical course characteristic of adrenomyeloneuropathy; mother and maternal grandmother are carriers; Southern blot analysis indicated that the donor subject has a deletion of exons 8-10 in the ATP-binding cassette, subfamily D, member 1 (ABCD1) gene.
